# Multiple horizontal transfers of a *Helitron* transposon associated with a parasitoid wasp

**DOI:** 10.1186/s13100-022-00278-y

**Published:** 2022-08-19

**Authors:** Pedro Heringer, Gustavo C. S. Kuhn

**Affiliations:** Departamento de Genética, Ecologia e Evolução, Instituto de Ciências Biológicas, Universidade Federal de Minas Gerais, MG CEP 31270-901 Belo Horizonte, Brazil

**Keywords:** *Helitron*, Transposon, Horizontal transfer

## Abstract

In a previous study we described a *Helitron* transposon that apparently became one of the segments in the symbiotic *Cotesia vestalis* bracovirus (CvBV) from the parasitoid wasp *C. vestalis*. We presented evidence that this *Helitron*, named Hel_c35, invaded the *C. vestalis* genome through a horizontal transfer (HT) event from a dipteran and was later transferred horizontally from *C. vestalis* to a lepidopteran species. Based on the phylogeny of Hel_c35, we suggested that both HTs occurred in East Asia. We have also anticipated that, as more sequenced genomes from new species become available, more HTs involving Hel_c35 would be detected. Although the inclusion of Hel_c35 as a CvBV segment turned out to be a methodological artifact, the fact that Hel_c35 copies are present in the genomes of *C. vestalis* and other arthropods still remains. Here, we investigated the evolution of Hel_c35 in arthropods using an updated data set to reassess our previous findings. Most species (95%) included in the present work had their genomes sequenced after our initial study was published, thus representing new descriptions of taxa harboring Hel_c35. Our results expand considerably the number of putative HTs involving Hel_c35, with up to dozens of previously undescribed events, and suggest that the most recent HTs associated with *C. vestalis* took place in Europe. Considering the phylogenetic distribution of Hel_c35, and the evidence that its DNA sequences are present in the calyx fluid of *C. vestalis* and tissues from its parasitized host, we argue that many HT events were favored by the behavior of this wasp.

**Supplementary Information:**

The online version contains supplementary material available at 10.1186/s13100-022-00278-y.

## Introduction

Horizontal Transfer (HT) events are defined as the exchange of DNA segments between organisms without the involvement of vertical inheritance from parent to offspring. Although HTs are major drivers of evolutionary change in prokaryotes, they are considerably less frequent in eukaryotes, especially in multicellular organisms. Nonetheless, more recently, HTs have been increasingly accepted as important events during the evolution of eukaryotes, promoting gene exchanges between distant-related taxa and facilitating the gain of adaptative functions in this domain of life (reviewed in [12, 14, 25, 27].

In contrast to most genomic components, transposons are DNA segments capable of moving from a locus to another and are found in multiple copies scattered across most eukaryotic genomes. These features make transposons one of the genetic entities most likely to be involved in successful HTs among eukaryotes. Indeed, as the number of eukaryotic sequenced genomes has grown considerably in the last few decades, the described examples of horizontal transposon transfers (HTTs) between eukaryotes have also increased, as well as the availability of new bioinformatic methods to detect those events [24, 28].

We previously described a *Helitron* transposon from the parasitoid wasp *Cotesia vestalis*, which was found to represent one of the circular segments of the symbiotic virus *C. vestalis* bracovirus (CvBV) [10]. We named this *Helitron* Hel_c35, as it was initially characterized from the segment 35 (GenBank accession: HQ009558.1) of the first complete CvBV genome assembly [4]. More recently, an update of the CvBV genome reduced its number of actual segments from 35 to 30 [29]. Among the excluded segments was the Hel_c35 sequence (segment 35), indicating that, in contrast to our previous suggestion, this *Helitron* is not part of the CvBV genome and thus its DNA sequences are not enclosed in viral particles. Although the inclusion of segment 35 in the initial version of CvBV genome most likely derived from an assembly artifact, it is not clear why Hel_c35 was isolated with the purified viral DNA and even found to be one of the most abundant segments of this bracovirus [4]. In spite of this revision, Hel_c35 copies are present in the *C. vestalis* genome, including a region with sequences conserved in proviral loci from bracoviruses [10]. In light of the current evidence, this insertion close to CvBV sequences is circumstantial and is not associated with the production of viral segments.

The first described Hel_c35 element has 5,294 bp and appears to be autonomous, containing a 4,538 bp gene encoding its transposase (GenBank accession: AEE09607.1) consisting of 1,384 amino acids. Furthermore, in our previous work, we showed that, not only this *C. vestalis Helitron* originated after a HTT event (probably from a *Drosophila* species to *C. vestalis*), but also was later transferred horizontally from *C. vestalis* to the domestic silk moth (*Bombyx mori*). Those HTTs probably occurred in East Asia and were likely facilitated by the close interaction that exists between *C. vestalis* and its potential hosts, which is a fundamental part of this wasp’s life cycle. However, we anticipated in our study that any HT description could be interpreted differently in the future as more species with sequenced genomes become available [10].

Here, we reassessed our earlier propositions using an updated data set that includes genomes sequenced more recently (in the last four years), providing a larger and more diverse sample of species. Our results reveal that Hel_c35 elements can be found in a considerably wider range of arthropod species from different orders than it was previously suggested. Likewise, our analysis indicates that Hel_c35 sequences in a large number of species are the result of multiple additional HT events, which are probably correlated with the parasitoid behavior of this wasp. In particular, the investigation of sequences more similar to Hel_c35 elements from *C. vestalis* suggests that several recent putative HTs took place in Europe.

## Results and discussion

We searched for Hel_c35 homologs with Blastn (hits with > 80% identity covering > 70% of the query) in all arthropod genomes available on GenBank [23] using the Hel_c35 sequence (GenBank accession: HQ009558.1) as a query. After excluding individual matches covering < 70% of the query length, a total of 285 sequences from 117 species were selected for further analyses (Table S1). Although the vast majority of taxa consisted of Lepidoptera species, several different insect orders and two spider species were found to harbor Hel_c35 sequences.

**Fig. 1:**
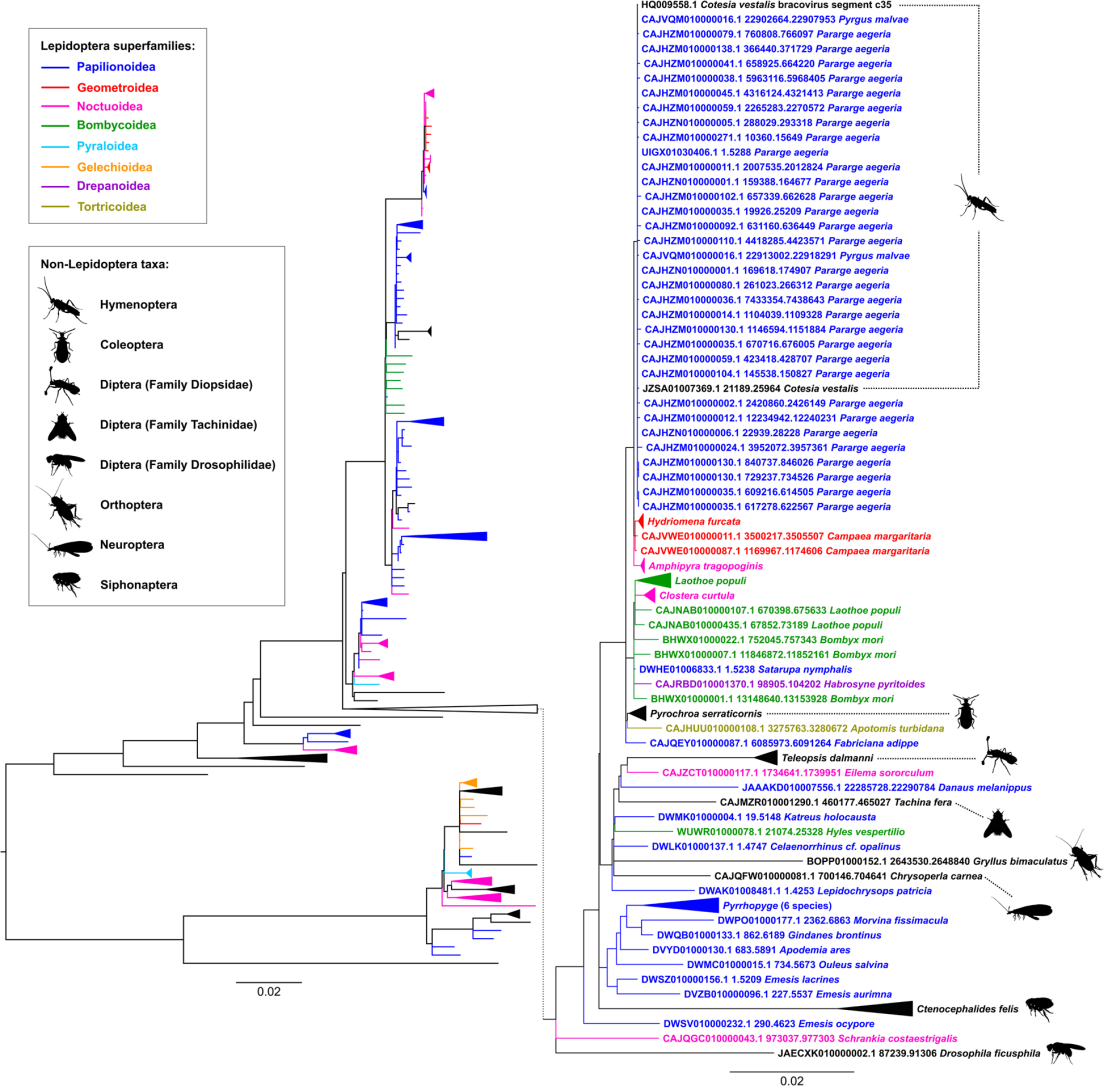
Phylogeny of Hel_c35 sequences. A Maximum Likelihood phylogeny including all 285 Hel_c35 sequences retrieved from arthropod genomes is represented on the left. A clade containing sequences closely related to the *C. vestalis* Hel_c35 is featured on the right. Lepidoptera species from different superfamilies are represented by different colors. Non-lepidopteran arthropods are represented in black. Branches with < 0.7 SH-aLRT statistical support were collapsed. The same phylogeny with branch supports and all taxa names is shown on Fig. S1

We aligned all the retrieved Hel_c35 sequences and conducted a phylogenetic analysis using the Maximum Likelihood method (see Materials and methods for details). The resulting phylogeny shows that Hel_c35 sequences found in specific taxa are mostly scattered across different branches, instead of representing the overall topology expected from the evolutionary relationships between organisms (Fig. 1). In addition, although several lepidopterans from the same superfamily grouped together, many of those clades contain species from distinct families. At the same time, several taxa from the same family did not cluster more closely with each other, even though some of them were in superfamily-specific clades (Fig. S1, Table S1).

Despite the diversity and incongruent topology observed in the resulting phylogeny, all Hel_c35 sequences included have > 80% nucleotide sequence identity, what would place its earliest origin at ~ 33 million years ago (MYA), assuming that this transposon evolves neutrally. This divergence time is at least 15 times more recent than the one estimated for the split between arachnids and insects (> 500 MYA) [15] and several times more recent than the estimated time of divergence between most insect orders [21]. The patchy distribution of taxa, together with the marked deviation between observed and expected divergence times among sequences, strongly indicate that Hel_c35 has been involved in multiple HTT events during its evolution. Even if we only consider the topological incongruences among sequences from distinct arthropod classes and insect orders, more than a dozen HTs would be necessary to explain their phylogenetic distribution. However, if we also take into account the phylogenetic discrepancies involving species from different superfamilies this number might be at least three times larger (Fig. 1 and Fig. S1). It is important to mention that artifacts associated with contamination are unlikely to explain most HTTs indicated in our results, as the large diversity of sequences from arthropods in our data belong to different genome sequencing projects of 32 institutions and include species sampled in at least 35 countries from 6 continents (Table S1).

Given the large number of sequences included in our phylogeny, we decided to focus our analysis in the main clade containing the *C. vestalis* Hel_c35 sequences (zoomed in clade on Fig. 1). This well supported clade (SH-aLRT branch support = 0.95) (see also Fig. S1 for support values) contains species from seven insect orders, along with a variety of Lepidoptera species from six different superfamilies. Similarly to the phylogeny as a whole, most of this clade topology does not reflect the evolutionary relationships between species. Moreover, the estimated evolutionary distances between many sequences in this clade (Table S2) also strongly deviate from the expected divergence times between taxa. For example, the cat flea *Ctenocephalides felis* and *Drosophila ficusphila* were the two species with the largest number of pairwise differences per site (0.0751) between their Hel_c35 sequences. Using a conservative assumption of one generation per year for all species, this clade would have its origin at ~ 12.5 MYA, which strongly contrasts with the estimated divergence time between most taxa included in this clade. For instance, *C. felis* and *D. ficusphila* are estimated to have diverged > 200 MYA, and lepidopterans are estimated to have diverged from *Gryllus bimaculatus* (Orthoptera) > 300 MYA [15]. In both examples, if Hel_c35 had exclusively evolved neutrally and inherited vertically, no homologous Hel_c35 copies would be expected to be found between these groups. This contrasts strikingly with the observed nucleotide identity of > 92% between all sequences in this clade.

**Fig. 2:**
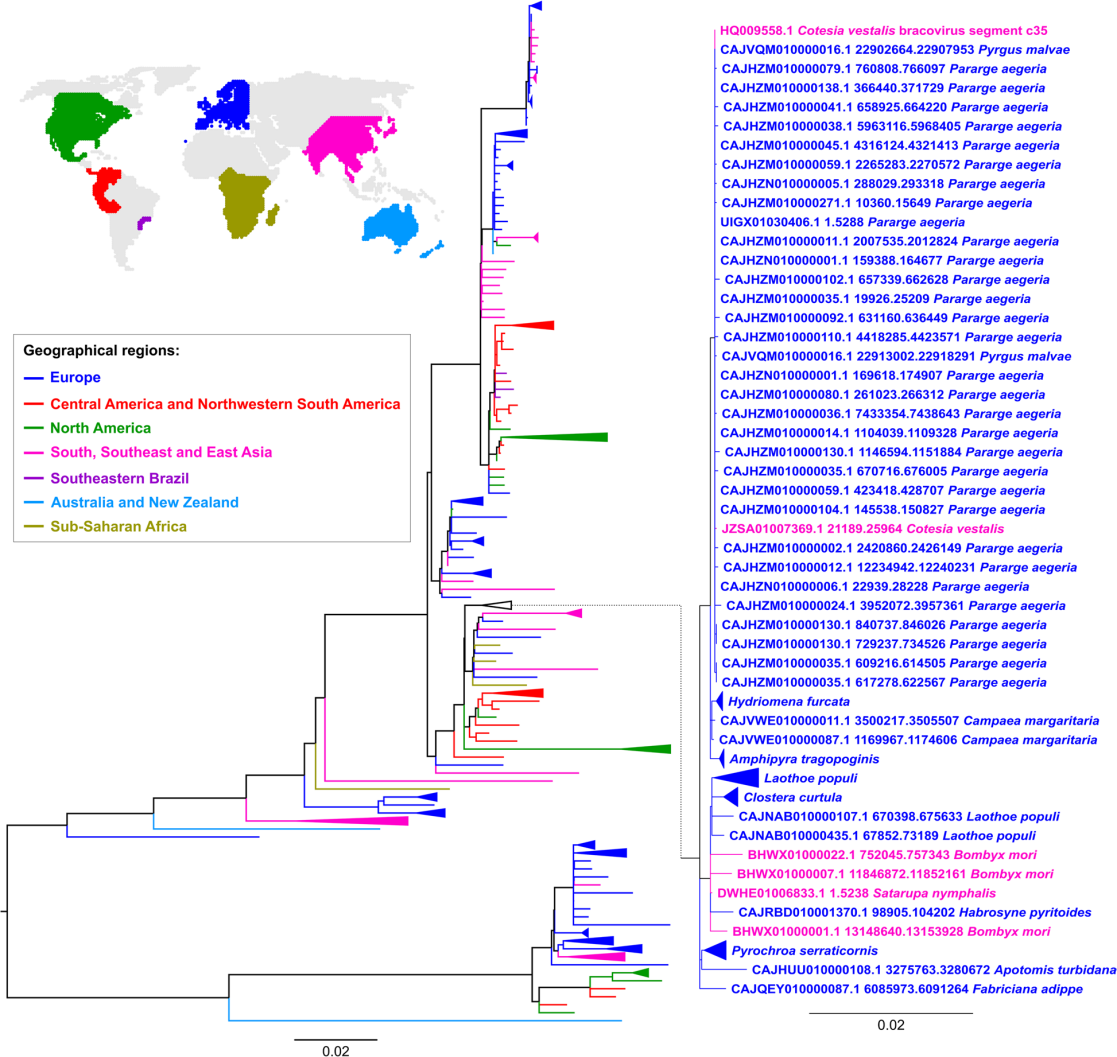
Geographical distribution of arthropod species containing Hel_c35. The same phylogeny of Hel_c35 sequences from Fig. 1 is represented, but with colors corresponding to the geographical location where the species were sampled (Table S1). A clade with species containing sequences closely related to CvBV Hel_c35 is featured expanded on the right. The same phylogeny with branch supports and all taxa names is shown on Fig. S2

A deviation from the expected pairwise nucleotide differences per site between species is even more pronounced in the clade comprising taxa with sequences more closely related to *C. vestalis* Hel_c35 elements (zoomed in clade on Fig. 2). All sequences in this proximal clade have > 99% identity between each other, even though they include species from three insect orders that diverged up to > 300 MYA (e.g., Hymenoptera and Lepidoptera) and six Lepidoptera superfamilies that diverged up to > 100 MYA (e.g., Bombycoidea and Tortricoidea) [15]. Considering the largest value of pairwise nucleotide differences per site among taxa in this clade, which is found between *Apotomis turbidana* and *Habrosyne pyritoides* (0.00983), its earliest origin would be ~ 1.64 MYA, in contrast to the estimated divergence times for some species included, which are longer by up to two orders of magnitude.

Some of the most conspicuous examples of recent HTTs are shown on the clade containing the Hel_c35 sequences from *C. vestalis*, *Pararge aegeria* and *Pyrgus malvae* (Fig. 1). The phylogenetic relationships between these three species are represented as a polytomy containing sequences with > 99.95% identity, what places its earliest date of origin at 0.068 MYA (68 thousand years ago). Considering that *P. aegeria* and *P. malvae* diverged > 70 MYA and these two Lepidoptera species have diverged from *C. vestalis* > 300 MYA, these values are at least three orders of magnitude higher than the maximum estimated divergence time for Hel_c35 sequences in this clade.

Even though the phylogenetic topology and level of identity between Hel_c35 sequences strongly suggest the existence of multiple HTTs, these events would also require some degree of geographic overlap between species to occur [19]. To verify if the geographical distribution of the analyzed species provides further evidence for HTT events, we represented our phylogeny by color coding the taxa according to the geographical locations where the species were sampled. Sample locations were assigned into one of seven regions defined by their biogeographic realm, bioregions and/or expected migration barriers. As expected, many phylogenetic incongruences (Fig. 1 and Fig. S1) involve taxa sampled in the same major geographical region (Fig. 2 and Fig. S2). For instance, considering 42 phylogenetic incongruencies that can be unambiguously assigned in our tree, 19 of these occur between species sampled within the same geographical region (Fig. S3).

Although we have considered the geographical proximity of samples in our analysis, it is still possible that more phylogenetic incongruences could be identified, as the sample location does not always correspond to the endemic region of a species, nor the whole extent of its geographical distribution. For example, the phylogenetic incongruence involving the lepidopteran *Chilo suppressalis* is not associated with a proximity between its sample region (East Asia) and the one for the closest branches (Europe) (Fig. S3). However, *C. suppressalis* can also be found in Europe [20]. Likewise, the phylogenetic incongruence observed for the cat flea *C. felis* is not associated with a geographical proximity between its sample and the ones in closely associated branches (Fig. S3). Nonetheless, *C. felis* is a species with a cosmopolitan distribution [22]. A similar case is observed for *C. vestalis* (see the next paragraph). Thus, the number of phylogenetic incongruences that involve species found in the same geographical region is probably larger than what is indicated from sample locations. Overall, many of the putative HTTs suggested by phylogenetic topological incongruences are further supported by a greater geographical proximity between the species involved.

Interestingly, 11 from the 14 species belonging to the *C. vestalis* Hel_c35 immediate clade correspond to samples from Europe (zoomed in clade on Fig. 2). From those 11 species, nine were sampled in the island of Great Britain (Table S1). The remaining two species were collected in Romania (*P. malvae* and *Fabriciana adippe*) but can also be found in Great Britain [2, 3]. Although the *C. vestalis* samples used in our analysis derive from East Asia (China and South Korea), this wasp species can also be found in several European countries [7], including Great Britain [1]. Hence, 12 out of 14 species in the immediate clade containing *C. vestalis* Hel_c35 sequences overlap geographically in Great Britain, indicating this island as the most probable region where the most recent HTT events involving *C. vestalis* Hel_c35 elements have occurred. It is worth mentioning that sequences from 11 out of 14 species in this immediate clade belong to assemblies provided by the Darwin Tree of Life Project (submitted to NCBI by the Wellcome Sanger Institute) (Table S1). This project focuses on sequencing genomes of a large number of organisms from Great Britain and Ireland with high quality assemblies [6]. Because a large number of insect genomes from Great Britain became recently available as a consequence of this project, species from this location could be overrepresented in the *C. vestalis* Hel_c35 immediate clade. However, although our results point to Great Britain as the location where the most recent events occurred according to the available data, the future genome sequencing of more species from different regions could reveal a different scenario.

Although it is difficult to infer the direction of HTTs, the diversity of Lepidoptera superfamilies at the base of most clades suggests that species in this order are the earliest donors of horizontally transferred Hel_c35 sequences. However, considering the large number of potential HTTs in the presented phylogeny, it is also possible that Lepidoptera species could have received Hel_c35 sequences by secondary HTT events (e.g., a HTT from a lepidopteran to a dipteran, which later transferred this transposon to other Lepidoptera species). The diversity and broad distribution of dipterans in the phylogeny (Fig. 1 and Fig. S1) indicate that species from this order were also donors of Hel_c35 elements. Nonetheless, because of mechanical and physiological constraints, direct HTs between insects should be considered rare events. In those cases, it is reasonable to expect the involvement of species like *C. vestalis* as likely HT vectors or intermediates, due to their life history which is thought to facilitate those events [24, 28]. That is probably the case for putative HTTs involving sequences more closely related to *C. vestalis* Hel_c35 elements (Fig. 1).

As mentioned previously, the DNA circle identified as segment 35 in the first CvBV genome assembly [4], which represents the Hel_c35 sequence, in fact does not constitute a viral segment and is not in the updated CvBV genome [29]. However, even though Hel_c35 is not part of CvBV, the fact that a large amount of DNA sequences corresponding to this transposon was identified in the calyx fluid of *C. vestalis* [4] suggests that Hel_c35 could be injected into hosts when this parasitoid wasp lays its eggs. To further test this hypothesis, we verified if reads corresponding to Hel_c35 sequences could be detected in the raw genome data of hemocytes from *Plutella xylostella* larvae parasitized by *C. vestalis* [29]. Blastn searches against the corresponding experiments in the NCBI SRA database (accession numbers: SRR11526873, SRR11537818 and SRR11537820) revealed thousands of reads (up to 5,000) in each SRA accession with > 99% sequence identity mapping to the whole Hel_c35 sequence (Fig. S4).

To confirm that these reads correspond to exogenous DNA injected by *C. vestalis* and not Hel_c35 elements from the *P. xylostella* genome, we also searched Hel_c35 sequences in the *P. xylostella* NCBI WGS database using Blastn. In this case, the best hits had < 67% sequence identity covering only up to 18% of the query (Fig. S4), indicating that *P. xylostella* does not have Hel_c35 elements in its genome. It is unlikely that this absence is the result of sequencing or assembly artifacts, as the WGS database contains nine *P. xylostella* genome assemblies associated with different BioProjects/BioSamples, variable levels of coverage and includes distinct sequencing platforms. For instance, we also verified if Hel_c35 could be detected in the raw data (SRA accession numbers: ERR7220502-ERR7220505) from the most recent *P. xylostella* genome assembly (WGS accession: CAKOAA01) using Blastn. Although a small number of hits from these searches had > 99% sequence identity with Hel_c35, most reads had < 90% sequence identity and mapped to the query with a patchy distribution (Fig. S4), which confirms the lack of Hel_c35 elements in the *P. xylostella* genome. Hence, the presence of Hel_c35 DNA in the calyx fluid of *C. vestalis* [4] and hemocytes from parasitized *P. xylostella* larvae [29] suggests that Hel_c35 elements are replicated in calyx cells and can be injected into tissues from hosts attacked by *C. vestalis*. These results also indicate a preferred direction for HTT events involving sequences more closely related to *C. vestalis* Hel_c35, which is from the parasitoid wasp to other species.

**Fig. 3:**
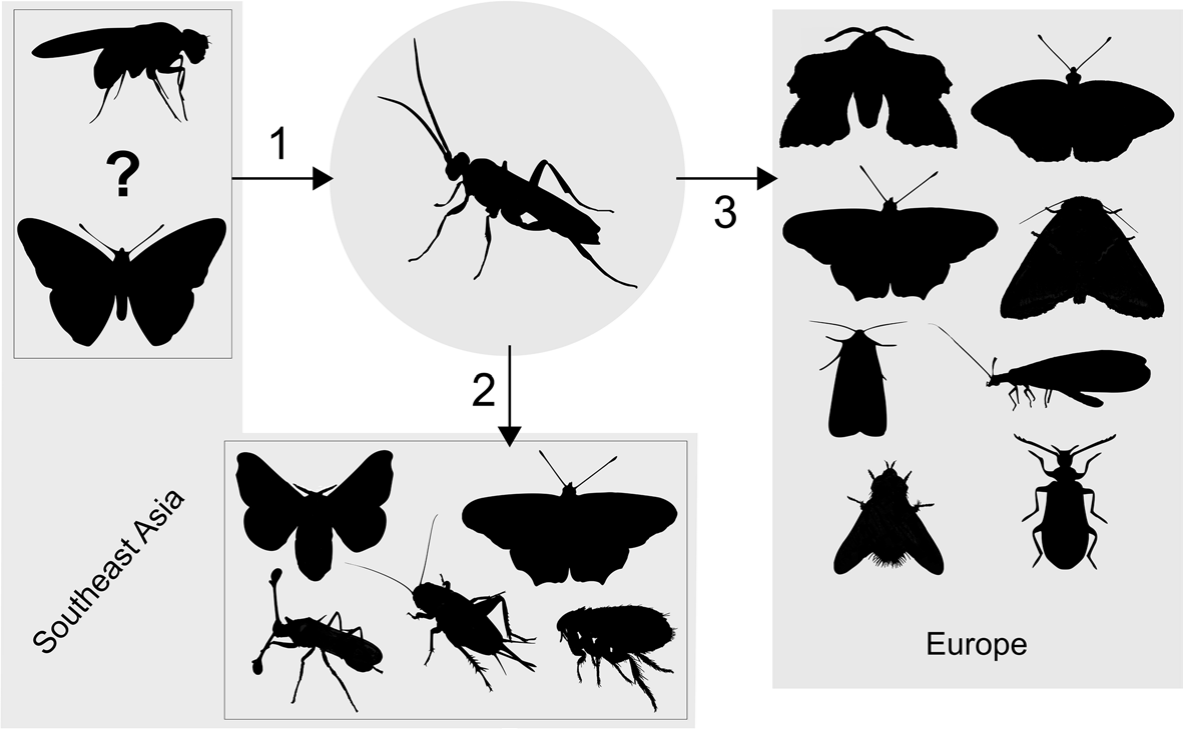
Hypothesis for HTTs involving Hel_c35 sequences closely related to the one found in *C. vestalis*. Arrows represent the probable direction of HTTs and numbers indicate the order which most HTTs events in each geographical region occurred. The earliest event from a Diptera or Lepidoptera species to *C. vestalis* (1) was followed by HTTs from *C. vestalis* to multiple insects from several orders, initially to species found in Southeast Asia (2) and more recently to species from Europe (3). Although most HTTs in 2 appear to have occurred earlier than those in 3, some European species are interspersed with, or more basal in relation to some Southeast Asian species, indicating that this chronological division is not clear cut

Considering the topology revealed by our phylogenetic analysis, the geographical distribution of the species and their natural history, we suggest the following hypothesis to explain the putative HTTs involving sequences more closely related to *C. vestalis* Hel_c35 elements. The originally Palearctic/eastern Asian distribution of *C. vestalis* [11] and several other lepidopterans and dipterans harboring closely related Hel_c35 sequences [10] indicates that *C. vestalis* acquired Hel_c35 by HT from an insect species within those orders, less than 12.5 MYA. In our previous work [10] we suggested a drosophilid as the most probable donor of the *C. vestalis* Hel_c35, given the evidence available at the time. Although our current result showing eastern Asian drosophilids near the base of the *C. vestalis* Hel_c35 clade provide some support for that scenario, it is not possible to reject that lepidopterans from the same geographical region could also have been potential donors. In any case, after this initial HTT event, Hel_c35 copies were transferred horizontally from *C. vestalis* to multiple species from several insect orders in different geographical locations, probably facilitated by the parasitoid behavior of this wasp (Fig. 3).

Lepidoptera species are overrepresented in our phylogeny, what could indicate a genome sequencing bias favoring this order. On the other hand, this could likewise be a consequence of lepidopterans being more frequently attacked by parasitoid wasps. This feature might be particularly relevant to explain the putative HTTs indicated in the immediate clade containing the *C. vestalis* Hel_c35 sequences (Fig. 1). Despite being considered a specialist parasitoid of the diamondback moth (*Plutella xylostella*), *C. vestalis* is known to attack lepidopterans from at least ten different families within eight superfamilies [11]. In view of the high diversity of lepidopteran larvae that can be targeted by *C. vestalis*, it is reasonable to expect that unspecific attacks to larvae from other insect orders could also occur in some conditions, even if rarely. In fact, the diversity of insect orders found in the main clade containing *C. vestalis* sequences in itself might be considered as evidence for the occurrence of those unspecific attacks. As we previously suggested [10], the detection of HTs involving parasitoid wasps and species outside the known range of hosts targeted by those wasps could be used to indicate potential cryptic interactions to be confirmed in future ecological and behavioral studies.

Overall, there are some important differences between the data presented here and the one presented in our previous work [10]. In our initial analysis, the single best hits from 24 species were included, as opposed to the current study, in which 285 sequences from 117 species were used, even though we used a more stringent selection criterion. For instance, here we considered the same minimum query coverage (> 70%) and identity (> 80%) as previously, but using the whole Hel_c35 (5,294 bp) as a reference, as opposed to a region of ~ 838 bp only containing the Rep coding sequence. The sampled species in our former analysis belonged to five insect orders, with one spider species, in contrast to the current sample that comprises 117 species from eight insect orders and two spider families. Therefore, not only the resulting data set presented here is larger, but is also more diverse. It is also worthwhile mentioning that using this more stringent sequence selection criterion, only five out of 24 species from the previous analysis were included in the current study. On the other hand, one of the new species included in the present data set (*Heliconius wallacei*) had its genome sequence already available before the previous study was conducted (November 2015), although we cannot explain the reason for its absence. The remaining 111 new species all had their genome sequences made available only after our previous work was submitted (September 2017), thus representing 95% of the current data set (Table S1).

The larger number of species in the present analysis reveals a more complex scenario regarding the evolutionary history of Hel_c35 sequences more closely related to the one found in *C. vestalis*. We previously suggested that East/Southeast Asia was probably the geographical region in which the most recent HTTs of Hel_c35 involving *C. vestalis* had occurred [10]. Although the evidence provided here still is consistent with a scenario in which the *C. vestalis* Hel_c35 originated from a HTT that probably occurred in East/Southeast Asia < 12.5 MYA, our current results add that this *Helitron* was probably horizontally transferred more recently to multiple insect species in Europe in the last few million years. Furthermore, our results presented here confirm the previous hypothesis that, as more genome sequencing projects would become available, new HT events would also probably be detected, resulting in different and more complete interpretations about the evolution of Hel_c35.

Given the large amount of putative HTTs involving *C. vestalis* as a donor of Hel_c35 sequences, and the evidence that Hel_c35 DNA copies are found in the calyx of *C. vestalis*, the future sequencing of DNAs present in the calyx fluid of *C. vestalis* from different lineages and geographical locations will be essential to confirm our proposed scenario. For instance, if extrachromosomal Hel_c35 copies turn out to be absent in European lineages of *C. vestalis*, our main hypotheses regarding the direction and geographical location of the most recent HTTs would be refuted, at least partially. Nevertheless, based on the current results, we suggest that at least some HTTs of Hel_c35 elements have been promoted by the replication and release of this *Helitron* in the calyx lumen of *C. vestalis*, followed by their injection in parasitized hosts.

## Materials and methods

We searched Hel_c35 homologs with Blastn in all arthropod genomes available (as in October 2021) on the Whole Genome Shotgun (WGS) contigs database from GenBank [23] using the first described Hel_c35 sequence (HQ009558.1) as a query. In order to include only highly similar elements in our analysis, we downloaded all Blastn aligned sequences from hits with > 80% sequence identity covering > 70% of the query. Those downloaded hits are sometimes composed by multiple separate matches which, together, cover > 70% of the query, instead of continuous sequences with the minimum query cover size. Hence, we adapted a Biopython [5] script to include only sequences covering > 70% (3,705 bp) of the query, and also to edit sequence descriptions as to keep only the hit accession number, sequence match range and the species name (Data S1). The resulting 285 sequences (Data S2) were aligned using the E-INS-i method in the MAFFT online service [13]. For the phylogenetic analysis, the best-fit evolutionary model (GTR + G + I) was selected using the Smart Model Selection (SMS) in PhyML [17]. The maximum likelihood phylogeny of sequences was inferred using the best topology from NNI and SPR methods, six random plus one parsimony starting trees and 10 substitution rate categories across sites, modelled with estimated gamma-shaped distribution parameter and a proportion of invariant sites. Branch supports were estimated using the approximate likelihood ratio test (aLRT) with the nonparametric Shimodaira–Hasegawa correction (SH-aLRT). The phylogenetic analysis procedures described above were conducted on PhyML 3.1 [9]. All branches with < 0.7 SH-aLRT statistical support were collapsed using TreeGraph 2 [26], with the final tree edited and visualized using FigTree v.1.4.2 (http://tree.bio.ed.ac.uk/software/figtree/; last accessed December 15, 2022). The species taxonomy and sample collection locations were obtained from their corresponding accession on GenBank [23], and additional information about the geographical distribution of organisms included in our analysis was obtained from various Web sources. The average nucleotide differences per site between groups in the main clade containing the *C. vestalis* Hel_c35 (Table S2) was calculated using MEGA X [16], and their divergence time estimated using the equation:

$$T=\frac{K}{2r}$$

where $T$ is the number of generations, $K$ is the number of substitutions per site, and $r$ is the nucleotide substitution rate. We considered that $r$ is equal to the mutation rate (*μ*), as expected for neutral mutations [8], and a value of *μ* equal to 3.0 × 10^–9^ for insect species [18]. To obtain a conservative estimation for the maximum time of divergence between sequences we considered one generation per year for all insect species, which is below the values commonly found for most taxa included in our analysis (usually from two up to several generations per year, depending on the order). Hence, in our estimates, the value found for $T$ is equal to the divergence time between species given in number of years.

## Supplementary Information

**Additional file 1: Table S1.** Information of all taxa included in the analyses. **Table S2.** Average base differences per site between groups in the main clade containing *C. vestalis* Hel_c35 elements. **Figure S1.** Same Maximum Likelihood phylogeny as Fig. 1 (main text), displaying taxa names and branch support values. Distinct Lepidoptera superfamilies are represented by different colors and non-lepidopteran arthropods are represented in black. See Materials and methods for details of the phylogenetic inference procedures. **Figure S2.** Same Maximum Likelihood phylogeny as Fig. 2 (main text), displaying taxa names and branch support values. Colors correspond to geographical locations where the species were sampled (Table S1). **Figure S3.** Mirrored cladograms representing the phylogeny in Fig. 1 (left) and Fig. 2 (right). Branches with two black asterisks indicate phylogenetic incongruences according to taxonomic classification of species. Branches with black and red asterisks indicate phylogenetic incongruences associated with geographical proximity between sample locations of the taxa involved. Thus, black and red asterisks correspond to incongruences potentially explained by the geographical overlap of species, which is a feature required for the occurrence of HT events. Because the geographical region where a species was sampled does not always correspond to the whole extent of the species distribution, it is likely that more phylogenetic incongruences are associated with spatial overlapping of the taxa involved (see text). **Figure S4.** Graphic summary results of Blastn searches against *P. xylostella* sequencing data using Hel_c35 as a query. (A-C) Hits in the raw sequencing data from hemocytes of *P. xylostella* larvae parasitized by *C. vestalis* in three experiments. Reads mapped to Hel_c35 belonging to SRA accessions: (A) SRR11526873, (B) SRR11537818 and (C) SRR11537820. (D) Hits from searches against *P. xylostella* genome assemblies in the WGS database. (E) Reads in the raw data (SRA accession: ERR7220503) from a *P. xylostella* genome assembly (WGS accession: CAKOAA01) mapped to Hel_c35. Only the SRA run ERR7220503 from this dataset is shown as an example. See main text for further information. **Data S1.** Biopython script to only include sequences with > 70% (3705 bp) and to edit FASTA descriptions to contain only the hit accession number, the sequence match range and the species name. **Data S2.** List of sequences descriptions used in the analysis, with their accession number, match range and the species name.

## Data Availability

The data generated and analyzed during this study are included in this published article and its supplementary information files.

## Abbreviations

CvBV: *Cotesia vestalis* bracovirus
HT: Horizontal transfer
HTT: Horizontal transposon transfer
MYA: Million years ago
SH-aLRT: Approximate likelihood ratio test with nonparametric Shimodaira–Hasegawa correction
