# Supplementary material for: Multiple horizontal transfers of a Helitron transposon associated with a parasitoid wasp
Source: Mob DNA. 2022 Aug 19;13:20. doi: 10.1186/s13100-022-00278-y (PMC9389653; doi:10.1186/s13100-022-00278-y)
Supplement: Supplementary file 1 — Additional file 1: Table S1. Information of all taxa included in the analyses. Table S2. Average base differences per site between groups in the main clade containing C. vestalis Hel_c35 elements. Figure S1. Same Maximum Likelihood phylogeny as Fig. 1 (main text), displaying taxa names and branch support values. Distinct Lepidoptera superfamilies are represented by different colors and non-lepidopteran arthropods are represented in black. See Materials and methods for details of the phylogenetic inference procedures. Figure S2. Same Maximum Likelihood phylogeny as Fig. 2 (main text), displaying taxa names and branch support values. Colors correspond to geographical locations where the species were sampled (Table S1). Figure S3. Mirrored cladograms representing the phylogeny in Fig. 1 (left) and Fig. 2 (right). Branches with two black asterisks indicate phylogenetic incongruences according to taxonomic classification of species. Branches with black and red asterisks indicate phylogenetic incongruences associated with geographical proximity between sample locations of the taxa involved. Thus, black and red asterisks correspond to incongruences potentially explained by the geographical overlap of species, which is a feature required for the occurrence of HT events. Because the geographical region where a species was sampled does not always correspond to the whole extent of the species distribution, it is likely that more phylogenetic incongruences are associated with spatial overlapping of the taxa involved (see text). Figure S4. Graphic summary results of Blastn searches against P. xylostella sequencing data using Hel_c35 as a query. (A-C) Hits in the raw sequencing data from hemocytes of P. xylostella larvae parasitized by C. vestalis in three experiments. Reads mapped to Hel_c35 belonging to SRA accessions: (A) SRR11526873, (B) SRR11537818 and (C) SRR11537820. (D) Hits from searches against P. xylostella genome assemblies in the WGS database. (E) Reads in the raw da [file 13100_2022_278_MOESM1_ESM.pdf]

Additional file 1

Table S1. Information of all taxa included in the analyses.

Rank	Species	# of sequences	Geographical location	Submission Institution	Submission Date
Class Insecta					
Order Lepidoptera					
Superfamily Papilionoidea					
Family Nymphalidae					
	Pararge aegeria	31	Scotland/UK	Wellcome Sanger Institute/UK, Stockholm University/SWE	2021-01-28, 2018-08-08
	Fabriciana adippe	1	Romania	Wellcome Sanger Institute/UK	2021-04-15
	Heliconius wallacei	1	Peru	University of Cambridge/UK	2015-11-29
	Vanessa cardui	5	Scotland/UK	Wellcome Sanger Institute/UK	2021-02-13
	Dryas iulia	7	Costa Rica	Cornell University/USA	2021-06-28
	Danaus melanippus	1	India	Iridian Genomes/USA	2020-01-30
	Nymphalis polychloros	1	Spain	Wellcome Sanger Institute/UK	2021-02-13
Family Riodinidae					
	Apodemia ares	1	USA	Florida Museum of Natural History/USA	2021-05-03
	Emesis lacrines	1	Costa Rica	Florida Museum of Natural History/USA	2021-05-03
	Emesis aurimna	1	Costa Rica	Florida Museum of Natural History/USA	2021-05-03
	Emesis ocypore	1	Peru	Florida Museum of Natural History/USA	2021-05-03
	Emesis heterochroa	1	Peru	Florida Museum of Natural History/USA	2021-05-03
Family Papilionidae					
	Parnassius apollo	5	Germany, Italy	Florida Museum of Natural History/USA, Stockholm University/SWE	2021-05-03, 2021-06-20
	Parnassius imperator	1	China	Florida Museum of Natural History/USA	2021-05-03
	Parnassius smintheus	1	Canada	Florida Museum of Natural History/USA	2021-05-03
	Zerynthia polyxena	1	Italy	Florida Museum of Natural History/USA	2021-05-03
	Archon apollinus	1	Greece	Florida Museum of Natural History/USA	2021-05-03
	Protesilaus protesilaus	2	Peru	Florida Museum of Natural History/USA	2021-05-03
Family Lycaenidae					
	Curetis bulis	1	Myanmar	Florida Museum of Natural History/USA	2021-05-03
	Cyaniris semiargus	3	Romania	Wellcome Sanger Institute/UK	2021-01-25
	Lysandra coridon	2	Romania	Wellcome Sanger Institute/UK	2021-02-13
	Lycaena phlaeas	1	Scotland/UK	Wellcome Sanger Institute/UK	2021-03-17
	Aricia agestis	1	Romania	Wellcome Sanger Institute/UK	2021-01-25
	Lysandra bellargus	1	Spain	Wellcome Sanger Institute/UK	2021-03-17
	Lepidochrysops patricia	1	South Africa	Florida Museum of Natural History/USA	2021-05-03
	Eumaeus atala	7	USA	University of Texas Southwestern/USA	2021-03-02
Family Pieridae					
	Pieris rapae	3	Scotland/UK	Wellcome Sanger Institute/UK	2021-01-25
Family Hesperiidae					
	Pyrgus malvae	3	Romania	Wellcome Sanger Institute/UK	2021-07-21
	Satarupa nymphalis	1	China	Florida Museum of Natural History/USA	2021-05-03
	Gindanes brontinus	1	Costa Rica	Florida Museum of Natural History/USA	2021-05-03
	Pyrrhopyge telassa	1	Peru	Florida Museum of Natural History/USA	2021-05-03
	Pyrrhopyge sergius	1	Peru	Florida Museum of Natural History/USA	2021-05-03
	Pyrrhopyge hadassa	1	Peru	Florida Museum of Natural History/USA	2021-05-03
	Pyrrhopyge kelita	1	Peru	Florida Museum of Natural History/USA	2021-05-03
	Pyrrhopyge crida	1	Costa Rica	Florida Museum of Natural History/USA	2021-05-03
	Pyrrhopyge pelota	1	Bolivia	Florida Museum of Natural History/USA	2021-05-03
	Celaenorrhinus cf. opalinus	1	Kenya	Florida Museum of Natural History/USA	2021-05-03
	Katreus holocausta	1	Cameroon	Florida Museum of Natural History/USA	2021-05-03
	Morvina fissimacula	1	Costa Rica	Florida Museum of Natural History/USA	2021-05-03
	Ouleus salvina	1	Costa Rica	Florida Museum of Natural History/USA	2021-05-03
	Cecropterus casica	1	USA	Florida Museum of Natural History/USA	2021-05-03
	Mylon lassia	1	Costa Rica	Florida Museum of Natural History/USA	2021-05-03
	Eburuncus unifasciata	1	Panama	Florida Museum of Natural History/USA	2021-05-03
	Oxynetra roscius	1	Brazil	Florida Museum of Natural History/USA	2021-05-03
	Duroca duroca	1	Brazil	Florida Museum of Natural History/USA	2021-05-03
	Charidia lucaria	1	Peru	Florida Museum of Natural History/USA	2021-05-03
	Aurina azines	1	Guyana	Florida Museum of Natural History/USA	2021-05-03
	Mimia cf. chiapaensis	1	Ecuador	Florida Museum of Natural History/USA	2021-05-03
	Pythonides amaryllis	1	Costa Rica	Florida Museum of Natural History/USA	2021-05-03
	Zopyrion sandace	1	Mexico	Florida Museum of Natural History/USA	2021-05-03
	Mimoniades ocyalus	1	Brazil	Florida Museum of Natural History/USA	2021-05-03
	Dalla cyprius	1	Peru	Florida Museum of Natural History/USA	2021-05-03
	Signeta flammeata	1	Australia	Florida Museum of Natural History/USA	2021-05-03
	Erynnis tages	1	Romania	Wellcome Sanger Institute/UK	2021-01-25
	Ectomis octomaculata	1	Costa Rica	Florida Museum of Natural History/USA	2021-05-03
	Cecropterus confusis	1	USA	Florida Museum of Natural History/USA	2021-05-03
	Thymelicus sylvestris	6	England/UK	Wellcome Sanger Institute/UK	2021-07-21
	Piruna pirus	1	USA	Florida Museum of Natural History/USA	2021-05-03
	Timochares trifasciata	1	Costa Rica	Florida Museum of Natural History/USA	2021-05-03
	Autochton oryx	1	Ecuador	Florida Museum of Natural History/USA	2021-05-03
Superfamily Geometroidea					
Family Geometridae					
	Campaea margaritaria	2	England/UK	Wellcome Sanger Institute/UK	2021-08-18
	Hydriomena furcata	4	England/UK	Wellcome Sanger Institute/UK	2021-08-18
	Ectropis grisescens	9	China	Institute of Plant Physiology and Ecology/CHN	2021-03-22
Superfamily Noctuoidea					
Family Noctuidae					
	Amphipyra tragopoginis	2	England/UK	Wellcome Sanger Institute/UK	2021-02-13
	Griposia aprilina	1	England/UK	Wellcome Sanger Institute/UK	2021-09-30
	Atethmia centrago	4	England/UK	Wellcome Sanger Institute/UK	2021-03-17
	Mythimna ferrago	1	England/UK	Wellcome Sanger Institute/UK	2021-07-06
	Autographa pulchrina	1	England/UK	Wellcome Sanger Institute/UK	2021-04-14
	Autographa gamma	1	England/UK	Wellcome Sanger Institute/UK	2021-01-25
	Trichoplusia ni	1	USA	Cornell University/USA	2018-10-01
	Mamestra brassicae	2	Wales/UK	Wellcome Sanger Institute/UK	2021-01-25
	Sesamia nonagrioides	1	France	Paris-Saclay University/FRA	2021-04-13
Family Notodontidae					
	Clostera curtula	3	England/UK	Wellcome Sanger Institute/UK	2021-04-14
	Ptilodon capucinus	4	England/UK	Wellcome Sanger Institute/UK	2021-09-11
Family Erebidae					
	Eilema sororculum	1	England/UK	Wellcome Sanger Institute/UK	2021-09-24
	Spilosoma lubricipeda	3	England/UK	Wellcome Sanger Institute/UK	2021-02-13
	Euproctis similis	3	England/UK	Wellcome Sanger Institute/UK	2021-01-25
	Spilarctia lutea	11	England/UK	Wellcome Sanger Institute/UK	2021-09-18
	Schrankia costaestrigalis	1	England/UK	Wellcome Sanger Institute/UK	2021-04-14
	Arctia plantaginis	3	Finland?	University of Cambridge/UK	2020-04-10
	Lymantria monacha	6	England/UK	Wellcome Sanger Institute/UK	2021-01-25
	Lymantria dispar	5	Japan, China	Laval University/CAN	2021-05-04
Superfamily Bombycoidea					
Family Bombycidae					
	Bombyx mori	3	Japan	The University of Tokyo/JPN	2020-11-06
Family Sphingidae					
	Laothoe populi	7	England/UK	Wellcome Sanger Institute/UK	2021-02-13
	Hyles vespertilio	1	Italy	Max Planck Institute of Molecular Cell Biology and Genetics/DEU	2020-01-29
Family Saturniidae					
	Samia ricini	7	India*	Gakushuin University/JPN	2020-06-20
Superfamily Pyraloidea					
Family Crambidae					
	Chilo suppressalis	1	China	Huazhong Agricultural University/CHN	2019-01-08
	Chrysoteuchia culmella	2	England/UK	Wellcome Sanger Institute/UK	2021-07-06
Superfamily Gelechioidea					
Family Blastobasidae					

				Blastobasis lacticolella	15	England/UK	Wellcome Sanger Institute/UK	2021-01-25
				Blastobasis adustella	4	England/UK	Wellcome Sanger Institute/UK	2021-05-19
		Superfamily Drepanoidea						
			Family Drepanidae					
				Habrosyne pyritoides	1	England/UK	Wellcome Sanger Institute/UK	2021-05-11
		Superfamily Tortricoidea						
			Family Tortricidae					
				Apotomis turbidana	1	England/UK	Wellcome Sanger Institute/UK	2021-01-25
	Order Diptera							
		Superfamily Diopsoidea						
			Family Diopsidae					
				Teleopsis dalmanni	4	Malaysia	SUNY Geneseo/USA, University of Maryland/USA	2020-09-23, 2020-10-30
		Superfamily Syrphoidea						
			Family Syrphidae					
				Cheilosia vulpina	1	England/UK	Wellcome Sanger Institute/UK	2021-09-30
				Melanostoma mellinum	3	England/UK	Wellcome Sanger Institute/UK	2021-09-11
		Superfamily Tephritoidea						
			Family Tephritidae					
				Bactrocera dorsalis	1	USA	Agricultural Research Service-USDA/USA	2014-12-03
		Superfamily Ephydroidea						
			Family Drosophilidae					
				Drosophila biarmipes	7	India to SE Asia*	University of Pennsylvania/USA	2019-05-08
				Drosophila ficusphila	1	Taiwan	Stanford University/USA	2021-04-28
				Drosophila auraria	1	Japan	University of California, Berkeley/USA	2019-08-21
				Drosophila bifasciata	1	Japan	University of California, Berkeley/USA	2019-11-15
				Drosophila obscura	3	Europe*, Serbia	National Institute of Genetics/JPN, Stanford University/USA	2017-10-14, 2021-04-28
				Drosophila ambigua	1	Serbia	Stanford University/USA	2021-04-28
				Drosophila guanche	1	Canary Islands/ESP	Centro Nacional de Análisis Genómico/ESP	2018-09-20
				Scaptomyza montana	2	USA*	Stanford University/USA	2021-06-16
				Scaptomyza flava	1	USA	University of California, Berkeley/USA	2018-12-17
		Superfamily Oestroidea						
			Family Tachinidae					
				Tachina fera	1	England/UK	Wellcome Sanger Institute/UK	2021-02-13
	Order Orthoptera							
		Superfamily Grylloidea						
			Family Gryllidae					
				Teleogryllus occipitalis	4	Japan	Waseda university/JPN	2020-02-22
				Gryllus bimaculatus	1	Japan	Tokushima University/JPN	2021-02-13
		Superfamily Eumastacoidea						
			Family Morabidae					
				Vandiemenella viatica	1	Australia	Uppsala University/SWE	2021-08-07
	Order Hymenoptera							
		Superfamily Ichneumonoidea						
			Family Braconidae					
				Cotesia vestalis	1	South Korea	Andong National University/KOR	2015-03-18
				Cotesia vestalis bracovirus segment c35	1	China	Zhejiang University/CHN	2011-05-09
			Family Ichneumonidae					
				Mesochorus sp.	1	Costa Rica	University of Georgia/USA	2021-06-16
	Order Coleoptera							
		Superfamily Tenebrionoidea						
			Family Pyrochroidae					
				Pyrochroa serraticornis	5	England/UK	Wellcome Sanger Institute/UK	2021-03-17
	Order Neuroptera							
			Family Chrysopidae					
				Chrysoperla carnea	1	England/UK	Wellcome Sanger Institute/UK	2021-04-14
	Order Siphonaptera							
		Superfamily Pulicoidea						
			Family Pulicidae					
				Ctenocephalides felis	5	USA	West Virginia University/USA	2018-08-24
	Order Phasmatodea							
			Family Phasmatidae					
				Clitarchus hookeri	1	New Zealand	Landcare Research/NZL	2017-11-16
Class Arachnida								
	Order Araneae							
		Superfamily Araneoidea						
			Family Nephilidae					
				Trichonephila inaurata madagascariensis	1	Madagascar	Institute for Advanced Biosciences - Keio University/JPN	2021-07-22
			Family Linyphiidae					
				Oedothorax gibbosus	1	Belgium	Royal Belgian Institute of Natural Sciences/BEL	2021-07-22

*Original or known distribution of the species (geographical location of biosample not available).

Table S2. Average base differences per site between groups in the main clade containing *C. vestalis* Hel_c35 elements.

	1	2	3	4	5	6	7	8	9	10	11	12	13	14	15	16	17	18	19	20	21	22	23	24	25	26	27	28	29	30	31	32	33	34	35	36	37	38	39	40
1.*Cotesia vestalis* bracovirus																																								
2.*Pararge aegeria*	0.0002																																							
3.*Pyrgus malvae*	0.0001	0.0003																																						
4.*Cotesia vestalis*	0.0002	0.0004	0.0003																																					
5.*Campaea margaritaria*	0.0011	0.0013	0.0012	0.0012																																				
6.*Hydriomena furcata*	0.0018	0.0020	0.0019	0.0021	0.0014																																			
7.*Amphipyra tragopoginis*	0.0022	0.0024	0.0023	0.0024	0.0019	0.0029																																		
8.*Pyrochroa serraticornis*	0.0043	0.0046	0.0044	0.0043	0.0044	0.0051	0.0054																																	
9.*Bombyx mori*	0.0061	0.0063	0.0062	0.0063	0.0063	0.0068	0.0072	0.0066																																
10.*Satarupa nymphalis*	0.0034	0.0036	0.0035	0.0035	0.0034	0.0041	0.0045	0.0040	0.0031																															
11.*Laothoe populi*	0.0063	0.0066	0.0065	0.0064	0.0063	0.0071	0.0074	0.0070	0.0061	0.0034																														
12.*Habrosyne pyritoides*	0.0061	0.0063	0.0061	0.0062	0.0063	0.0068	0.0071	0.0067	0.0057	0.0031	0.0061																													
13.*Fabriciana adippe*	0.0051	0.0053	0.0052	0.0050	0.0050	0.0058	0.0061	0.0058	0.0073	0.0048	0.0077	0.0074																												
14.*Clostera curtula*	0.0059	0.0061	0.0060	0.0058	0.0056	0.0066	0.0070	0.0065	0.0058	0.0028	0.0053	0.0058	0.0073																											
15.*Apotomis turbidana*	0.0072	0.0074	0.0073	0.0073	0.0067	0.0080	0.0079	0.0078	0.0096	0.0066	0.0093	0.0098	0.0090	0.0086																										
16.*Gindanes brontinus*	0.0146	0.0146	0.0145	0.0148	0.0142	0.0150	0.0153	0.0132	0.0159	0.0128	0.0154	0.0159	0.0157	0.0153	0.0143																									
17.*Apodemia ares*	0.0116	0.0116	0.0115	0.0113	0.0109	0.0123	0.0123	0.0112	0.0132	0.0104	0.0127	0.0130	0.0130	0.0122	0.0126	0.0090																								
18.*Pyrrhopyge telassa*	0.0134	0.0134	0.0133	0.0131	0.0126	0.0141	0.0142	0.0138	0.0149	0.0119	0.0149	0.0148	0.0148	0.0150	0.0170	0.0091	0.0096																							
19.*Pyrrhopyge sergius*	0.0160	0.0160	0.0159	0.0158	0.0157	0.0167	0.0167	0.0165	0.0176	0.0147	0.0177	0.0174	0.0174	0.0175	0.0197	0.0118	0.0120	0.0091																						
20.*Pyrrhopyge hadassa*	0.0166	0.0166	0.0165	0.0165	0.0161	0.0173	0.0173	0.0170	0.0182	0.0153	0.0182	0.0180	0.0180	0.0182	0.0199	0.0121	0.0128	0.0091	0.0071																					
21.*Pyrrhopyge kelita*	0.0173	0.0173	0.0172	0.0172	0.0168	0.0179	0.0182	0.0177	0.0189	0.0162	0.0195	0.0187	0.0185	0.0188	0.0201	0.0120	0.0133	0.0118	0.0081	0.0016																				
22.*Pyrrhopyge crida*	0.0160	0.0160	0.0159	0.0158	0.0153	0.0166	0.0167	0.0164	0.0178	0.0143	0.0176	0.0176	0.0174	0.0178	0.0193	0.0110	0.0124	0.0090	0.0074	0.0065	0.0082																			
23.*Pyrrhopyge pelota*	0.0201	0.0202	0.0200	0.0199	0.0204	0.0208	0.0211	0.0208	0.0215	0.0187	0.0217	0.0214	0.0216	0.0215	0.0241	0.0166	0.0171	0.0143	0.0123	0.0129	0.0140	0.0130																		
24.*Emesis lacrines*	0.0122	0.0122	0.0121	0.0121	0.0120	0.0127	0.0130	0.0110	0.0138	0.0111	0.0135	0.0139	0.0134	0.0133	0.0131	0.0121	0.0106	0.0120	0.0146	0.0152	0.0160	0.0147	0.0196																	
25.*Eilema sororculum*	0.0148	0.0148	0.0147	0.0150	0.0143	0.0153	0.0156	0.0142	0.0168	0.0138	0.0164	0.0169	0.0159	0.0162	0.0160	0.0175	0.0158	0.0170	0.0197	0.0201	0.0199	0.0195	0.0239	0.0156																
26.*Celaenorrhinus cf opalinus*	0.0116	0.0116	0.0115	0.0115	0.0108	0.0121	0.0121	0.0123	0.0136	0.0107	0.0135	0.0135	0.0131	0.0131	0.0140	0.0139	0.0121	0.0156	0.0177	0.0183	0.0195	0.0181	0.0229	0.0124	0.0114															
27.*Katreus holocausta*	0.0127	0.0129	0.0128	0.0127	0.0121	0.0136	0.0138	0.0128	0.0148	0.0115	0.0142	0.0149	0.0143	0.0137	0.0146	0.0152	0.0142	0.0171	0.0197	0.0201	0.0205	0.0191	0.0238	0.0147	0.0135	0.0108														
28.*Teleopsis dalmanni*	0.0262	0.0262	0.0261	0.0262	0.0260	0.0267	0.0268	0.0223	0.0280	0.0249	0.0265	0.0284	0.0275	0.0267	0.0209	0.0296	0.0270	0.0223	0.0246	0.0246	0.0235	0.0243	0.0274	0.0281	0.0236	0.0157	0.0253													
29.*Morvina fissimacula*	0.0209	0.0209	0.0208	0.0209	0.0207	0.0214	0.0219	0.0215	0.0228	0.0194	0.0228	0.0229	0.0225	0.0225	0.0243	0.0127	0.0168	0.0176	0.0198	0.0205	0.0221	0.0196	0.0256	0.0198	0.0234	0.0227	0.0239	0.0278												
30.*Ouleus salvina*	0.0164	0.0166	0.0165	0.0165	0.0166	0.0173	0.0175	0.0176	0.0183	0.0154	0.0185	0.0187	0.0183	0.0185	0.0203	0.0154	0.0139	0.0170	0.0193	0.0197	0.0192	0.0189	0.0235	0.0154	0.0207	0.0194	0.0208	0.0256	0.0247											
31.*Emesis aurimna*	0.0194	0.0194	0.0193	0.0197	0.0194	0.0199	0.0201	0.0189	0.0210	0.0178	0.0208	0.0207	0.0201	0.0202	0.0211	0.0192	0.0177	0.0203	0.0232	0.0237	0.0250	0.0232	0.0275	0.0140	0.0227	0.0209	0.0223	0.0361	0.0277	0.0238										
32.*Hyles vespertilio*	0.0156	0.0155	0.0154	0.0156	0.0151	0.0161	0.0165	0.0160	0.0173	0.0146	0.0177	0.0177	0.0167	0.0172	0.0181	0.0167	0.0154	0.0172	0.0205	0.0207	0.0224	0.0197	0.0257	0.0158	0.0146	0.0144	0.0154	0.0181	0.0266	0.0213	0.0246									
33.*Emesis ocypore*	0.0178	0.0178	0.0177	0.0179	0.0181	0.0183	0.0186	0.0182	0.0190	0.0161	0.0195	0.0194	0.0189	0.0187	0.0214	0.0185	0.0173	0.0194	0.0220	0.0224	0.0244	0.0217	0.0273	0.0176	0.0215	0.0211	0.0221	0.0257	0.0279	0.0236	0.0255	0.0242								
34.*Chrysoperla carnea*	0.0211	0.0213	0.0212	0.0211	0.0215	0.0218	0.0222	0.0216	0.0233	0.0205	0.0234	0.0238	0.0223	0.0230	0.0247	0.0238	0.0226	0.0245	0.0269	0.0276	0.0277	0.0272	0.0319	0.0216	0.0196	0.0203	0.0206	0.0247	0.0321	0.0283	0.0303	0.0229	0.0293							
35.*Tachina fera*	0.0221	0.0221	0.0220	0.0217	0.0215	0.0228	0.0230	0.0225	0.0235	0.0208	0.0233	0.0239	0.0235	0.0228	0.0251	0.0243	0.0230	0.0256	0.0285	0.0293	0.0309	0.0290	0.0335	0.0229	0.0210	0.0209	0.0215	0.0250	0.0338	0.0295	0.0314	0.0267	0.0318	0.0304						
36.*Lepidochrysops patricia*	0.0182	0.0184	0.0183	0.0180	0.0184	0.0189	0.0185	0.0187	0.0205	0.0177	0.0205	0.0210	0.0203	0.0198	0.0212	0.0210	0.0192	0.0207	0.0245	0.0248	0.0266	0.0236	0.0312	0.0196	0.0172	0.0174	0.0172	0.0226	0.0312	0.0265	0.0286	0.0225	0.0296	0.0288	0.0287					
37.*Schrankia costaestrigalis*	0.0268	0.0268	0.0267	0.0267	0.0259	0.0273	0.0276	0.0272	0.0278	0.0252	0.0283	0.0280	0.0275	0.0279	0.0294	0.0277	0.0268	0.0291	0.0315	0.0322	0.0338	0.0305	0.0368	0.0271	0.0294	0.0292	0.0298	0.0325	0.0373	0.0335	0.0354	0.0325	0.0309	0.0366	0.0403	0.0363				
38.*Drosophila ficusphila*	0.0416	0.0418	0.0417	0.0416	0.0416	0.0420	0.0425	0.0427	0.0438	0.0412	0.0441	0.0431	0.0434	0.0438	0.0448	0.0426	0.0419	0.0448	0.0468	0.0453	0.0470	0.0473	0.0525	0.0426	0.0448	0.0437	0.0446	0.0480	0.0539	0.0474	0.0501	0.0469	0.0446	0.0520	0.0564	0.0503	0.0503			
39.*Danaus melanippus*	0.0271	0.0273	0.0272	0.0272	0.0270	0.0279	0.0275	0.0265	0.0289	0.0265	0.0286	0.0293	0.0285	0.0281	0.0279	0.0297	0.0275	0.0299	0.0321	0.0323	0.0320	0.0316	0.0360	0.0282	0.0246	0.0243	0.0248	0.0386	0.0362	0.0328	0.0349	0.0289	0.0331	0.0343	0.0340	0.0318	0.0410	0.0557		
40.*Ctenocephalides felis*	0.0507	0.0507	0.0506	0.0508	0.0483	0.0510	0.0517	0.0508	0.0524	0.0499	0.0526	0.0530	0.0524	0.0519	0.0528	0.0530	0.0501	0.0534	0.0563	0.0561	0.0559	0.0562	0.0610	0.0507	0.0531	0.0531	0.0530	0.0566	0.0602	0.0567	0.0596	0.0544	0.0554	0.0605	0.0626	0.0552	0.0634	0.0751	0.0620	
41.*Gryllus bimaculatus*	0.0337	0.0337	0.0336	0.0319	0.0325	0.0332	0.0341	0.0324	0.0355	0.0329	0.0343	0.0355	0.0353	0.0350	0.0317	0.0370	0.0357	0.0347	0.0374	0.0377	0.0370	0.0371	0.0417	0.0317	0.0340	0.0300	0.0314	0.0472	0.0408	0.0386	0.0419	0.0322	0.0390	0.0379	0.0399	0.0350	0.0465	0.0623	0.0465	0.0676

Figure S1. Same Maximum Likelihood phylogeny as Fig. 1 (main text), displaying taxa names and branch support values. Distinct Lepidoptera superfamilies are represented by different colors and non-lepidopteran arthropods are represented in black. See Materials and Methods for details of the phylogenetic inference procedures.

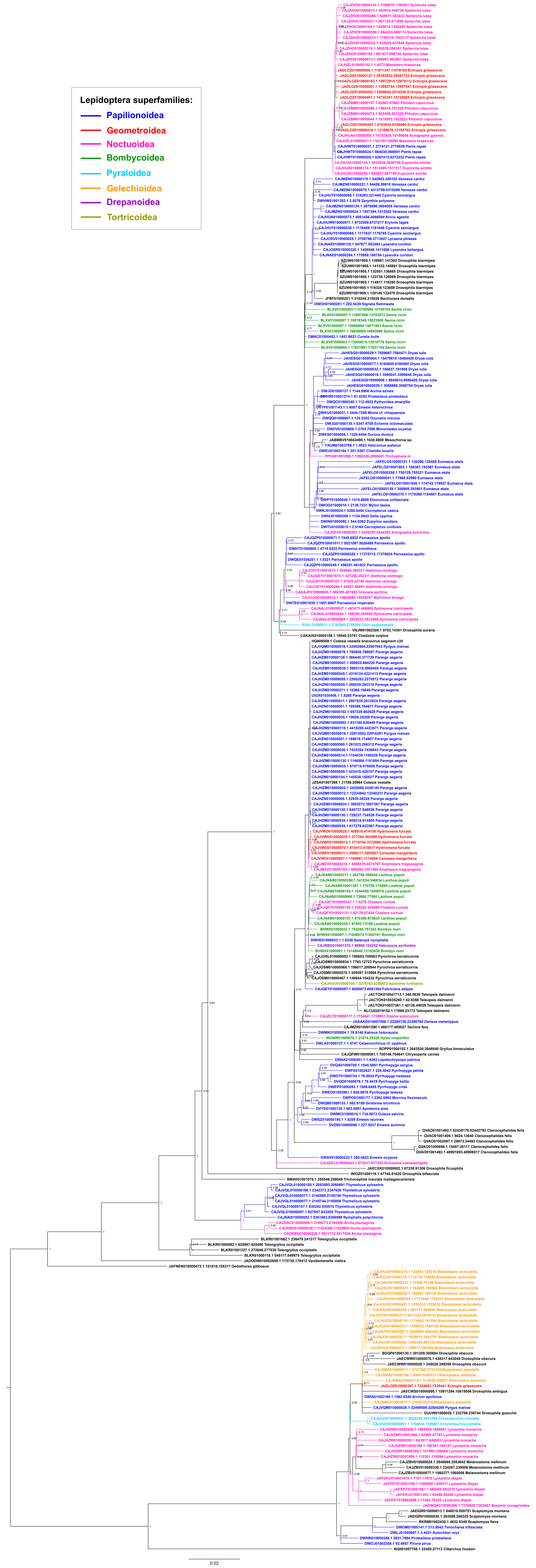

Lepidoptera superfamilies:
Papilionoidea
Geometroidea
Noctuoidea
Bombycoidea
Pyraloidea
Gelechioidea
Drepanoidea
Tortricoidea
0.02

Figure S2. Same Maximum Likelihood phylogeny as Fig. 2 (main text), displaying taxa names and branch support values. Colors correspond to geographical locations where the species were sampled (Table S1).

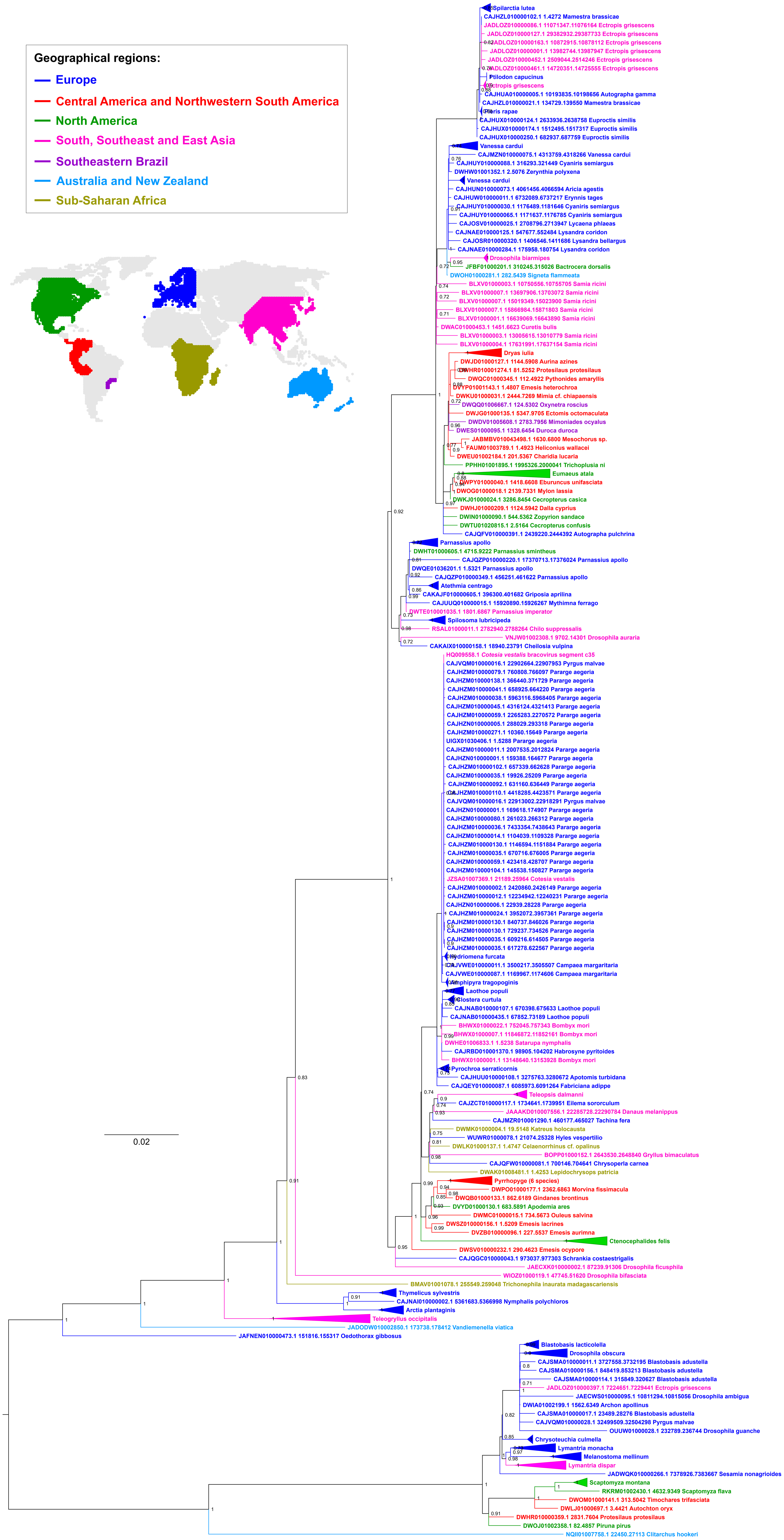

Geographical regions:
Europe
Central America and Northwestern South America
North America
South, Southeast and East Asia
Southeastern Brazil
Australia and New Zealand
Sub-Saharan Africa
Spilarctia lutea
CAJHZL010000102.1 1.4272 Mamestra brassicae
JADLOZ010000086.1 11071347.11076164 Ectropis grisescens
JADLOZ010000127.1 29382932.29387733 Ectropis grisescens
JADLOZ010000163.1 10872915.10878112 Ectropis grisescens
JADLOZ010000001.1 13982744.13987947 Ectropis grisescens
JADLOZ010000452.1 2509044.2514246 Ectropis grisescens
JADLOZ010000461.1 14720351.14725555 Ectropis grisescens
Ptilodon capucinus
Ectropis grisescens
CAJHUA010000005.1 10193835.10198656 Autographa gamma
CAJHZL010000021.1 134729.139550 Mamestra brassicae
Pieris rapae
CAJHUX010000124.1 2633936.2638758 Euproctis similis
CAJHUX010000174.1 1512495.1517317 Euproctis similis
CAJHUX010000250.1 682937.687759 Euproctis similis
Vanessa cardui
CAJMZN010000075.1 4313759.4318266 Vanessa cardui
CAJHUY010000088.1 316293.321449 Cyaniris semiargus
DWHW01001352.1 2.5076 Zerynthia polyxena
Vanessa cardui
CAJHUN010000073.1 4061456.4066594 Aricia agestis
CAJHUW010000011.1 6732089.6737217 Erynnis tages
CAJHUY010000030.1 1176489.1181646 Cyaniris semiargus
CAJHUY010000065.1 1171637.1176785 Cyaniris semiargus
CAJOSV010000025.1 2708796.2713947 Lycaena phlaeas
CAJNAE010000125.1 547677.552484 Lysandra coridon
CAJOSR010000320.1 1406546.1411686 Lysandra bellargus
CAJNAE010000284.1 175958.180754 Lysandra coridon
Drosophila biarmipes
JFBF01000201.1 310245.315026 Bactrocera dorsalis
DWOH01000281.1 282.5439 Signeta flammeata
BLXV01000003.1 10750556.10755705 Samia ricini
BLXV01000007.1 13697906.13703072 Samia ricini
BLXV01000007.1 15019349.15023900 Samia ricini
BLXV01000007.1 15866984.15871803 Samia ricini
BLXV01000001.1 16639069.16643890 Samia ricini
DWAC01000453.1 1451.6623 Curetis bulis
BLXV01000003.1 13005615.13010779 Samia ricini
BLXV01000004.1 17631991.17637154 Samia ricini
Dryas iulia
DWJD01000127.1 1144.5908 Aurina azines
DWHR010001274.1 81.5252 Protesilaus protesilaus
DWQC01000345.1 112.4922 Pythonides amaryllis
DVYP01001143.1 1.4807 Emesis heterochroa
DWKU01000031.1 2444.7269 Mimia cf. chiapaensis
DWQQ01006667.1 124.5302 Oxynetra roscius
DWJG01000135.1 5347.9705 Ectomis octomaculata
DWDV01005608.1 2783.7956 Mimoniades ocyalus
DWES01000095.1 1328.6454 Duroca duroca
JABMBV010043498.1 1630.6800 Mesochorus sp.
FAUM01003789.1 1.4923 Heliconius wallacei
DWEU01002184.1 201.5367 Charidia lucaria
PPHH01001895.1 1995326.2000041 Trichoplusia ni
Eumaeus atala
DWPY01000040.1 1418.6608 Eburuncus unifasciata
DWOG01000018.1 2139.7331 Mylon lassia
DWKJ01000024.1 3286.8454 Cecropterus casica
DWHJ01000209.1 1124.5942 Dalla cyprius
DWIN01000090.1 544.5362 Zopyrion sandace
DWTU01020815.1 2.5164 Cecropterus confusis
CAJQFV010000391.1 2439220.2444392 Autographa pulchrina
Parnassius apollo
DWHT01000605.1 4715.9222 Parnassius smintheus
CAJQZP010000220.1 17370713.17376024 Parnassius apollo
DWQE01036201.1 1.5321 Parnassius apollo
CAJQZP010000349.1 456251.461622 Parnassius apollo
Atethmia centrago
CAKAJF010000605.1 396300.401682 Griposia aprilina
CAJUUQ010000015.1 15920890.15926267 Mythimna ferrago
DWTE01001035.1 1801.6867 Parnassius imperator
Spilosoma lubricipeda
RSAL01000011.1 2782940.2788264 Chilo suppressalis
VNJW01002308.1 9702.14301 Drosophila auraria
CAKAIX010000158.1 18940.23791 Cheilosia vulpina
HQ009558.1 Cotesia vestalis bracovirus segment c35
CAJVQM010000016.1 22902664.22907953 Pyrgus malvae
CAJHZM010000079.1 760808.766097 Pararge aegeria
CAJHZM010000138.1 366440.371729 Pararge aegeria
CAJHZM010000041.1 658925.664220 Pararge aegeria
CAJHZM010000038.1 5963116.5968405 Pararge aegeria
CAJHZM010000045.1 4316124.4321413 Pararge aegeria
CAJHZM010000059.1 2265283.2270572 Pararge aegeria
CAJHZN010000005.1 288029.293318 Pararge aegeria
CAJHZM010000271.1 10360.15649 Pararge aegeria
UIGX01030406.1 1.5288 Pararge aegeria
CAJHZM010000011.1 2007535.2012824 Pararge aegeria
CAJHZN010000001.1 159388.164677 Pararge aegeria
CAJHZM010000102.1 657339.662628 Pararge aegeria
CAJHZM010000035.1 19926.25209 Pararge aegeria
CAJHZM010000092.1 631160.636449 Pararge aegeria
CAJHZM010000110.1 4418285.4423571 Pararge aegeria
CAJVQM010000016.1 22913002.22918291 Pyrgus malvae
CAJHZN010000001.1 169618.174907 Pararge aegeria
CAJHZM010000080.1 261023.266312 Pararge aegeria
CAJHZM010000036.1 7433354.7438643 Pararge aegeria
CAJHZM010000014.1 1104039.1109328 Pararge aegeria
CAJHZM010000130.1 1146594.1151884 Pararge aegeria
CAJHZM010000035.1 670716.676005 Pararge aegeria
CAJHZM010000059.1 423418.428707 Pararge aegeria
CAJHZM010000104.1 145538.150827 Pararge aegeria
JZSA01007369.1 21189.25964 Cotesia vestalis
CAJHZM010000002.1 2420860.2426149 Pararge aegeria
CAJHZM010000012.1 12234942.12240231 Pararge aegeria
CAJHZN010000006.1 22939.28228 Pararge aegeria
CAJHZM010000024.1 3952072.3957361 Pararge aegeria
CAJHZM010000130.1 840737.846026 Pararge aegeria
CAJHZM010000130.1 729237.734526 Pararge aegeria
CAJHZM010000035.1 609216.614505 Pararge aegeria
CAJHZM010000035.1 617278.622567 Pararge aegeria
Hydriomena furcata
CAJVWE010000011.1 3500217.3505507 Campaea margaritaria
CAJVWE010000087.1 1169967.1174606 Campaea margaritaria
Amphipyra tragopoginis
Laothoe populi
Clostera curtula
CAJNAB010000107.1 670398.675633 Laothoe populi
CAJNAB010000435.1 67852.73189 Laothoe populi
BHWX01000022.1 752045.757343 Bombyx mori
BHWX01000007.1 11846872.11852161 Bombyx mori
DWHE01006833.1 1.5238 Satarupa nymphalis
CAJRBD010001370.1 98905.104202 Habrosyne pyritoides
BHWX01000001.1 13148640.13153928 Bombyx mori
Pyrochroa serraticornis
CAJHUU010000108.1 3275763.3280672 Apotomis turbidana
CAJQEY010000087.1 6085973.6091264 Fabriciana adippe
Teleopsis dalmanni
CAJZCT010000117.1 1734641.1739951 Eilema sororculum
JAAAKD010007556.1 22285728.22290784 Danaus melanippus
CAJMZR010001290.1 460177.465027 Tachina fera
DWMK01000004.1 19.5148 Katreus holocausta
WUWR01000078.1 21074.25328 Hyles vespertilio
DWLK01000137.1 1.4747 Celaenorrhinus cf. opalinus
BOPP01000152.1 2643530.2648840 Gryllus bimaculatus
CAJQFW010000081.1 700146.704641 Chrysoperla carnea
DWAK01008481.1 1.4253 Lepidochrysops patricia
Pyrrhopyge (6 species)
DWPO01000177.1 2362.6863 Morvina fissimacula
DWQB01000133.1 862.6189 Gindanes brontinus
DVYD01000130.1 683.5891 Apodemia ares
DWMC01000015.1 734.5673 Ouleus salvina
DWSZ010000156.1 1.5209 Emesis lacrines
DVZB010000096.1 227.5537 Emesis aurimna
Ctenocephalides felis
DWSV010000232.1 290.4623 Emesis ocypore
CAJQGC010000043.1 973037.977303 Schrankia costaestrigalis
JAECXK010000002.1 87239.91306 Drosophila ficusphila
WIOZ01000119.1 47745.51620 Drosophila bifasciata
BMAV01001078.1 255549.259048 Trichonephila inaurata madagascariensis
Thymelicus sylvestris
CAJNAI010000002.1 5361683.5366998 Nymphalis polychloros
Arctia plantaginis
Teleogryllus occipitalis
JADODW010002850.1 173738.178412 Vandiemenella viatica
JAFNEN010000473.1 151816.155317 Oedothorax gibbosus
Blastobasis lacticolella
Drosophila obscura
CAJSMA010000011.1 3727558.3732195 Blastobasis adustella
CAJSMA010000156.1 848419.853213 Blastobasis adustella
CAJSMA010000114.1 315849.320627 Blastobasis adustella
JADLOZ010000397.1 7224651.7229441 Ectropis grisescens
JAECWS010000095.1 10811294.10815056 Drosophila ambigua
DWIA01002199.1 1562.6349 Archon apollinus
CAJSMA010000017.1 23489.28276 Blastobasis adustella
CAJVQM010000028.1 32499509.32504298 Pyrgus malvae
OUUW01000028.1 232789.236744 Drosophila guanche
Chrysoteuchia culmella
Lymantria monacha
Melanostoma mellinum
Lymantria dispar
JADWQK010000266.1 7378926.7383667 Sesamia nonagrioides
Scaptomyza montana
RKRM01002430.1 4632.9349 Scaptomyza flava
DWOM01000141.1 313.5042 Timochares trifasciata
DWLJ01000697.1 3.4421 Autochton oryx
DWHR010000359.1 2831.7604 Protesilaus protesilaus
DWQJ01002358.1 82.4857 Piruna pirus
NQII01007758.1 22450.27113 Clitarchus hookeri
0.02

Figure S3. Mirrored cladograms representing the phylogeny in Fig. 1 (left) and Fig. 2 (right). Branches with two black asterisks indicate phylogenetic incongruences according to taxonomic classification of species. Branches with black and red asterisks indicate phylogenetic incongruences associated with geographical proximity between sample locations of the taxa involved. Thus, black and red asterisks correspond to incongruences potentially explained by the geographical overlap of species, which is a feature required for the occurrence of HT events. Because the geographical region where a species was sampled does not always correspond to the whole extent of the species distribution, it is likely that more phylogenetic incongruences are associated with spatial overlapping of the taxa involved (see text).

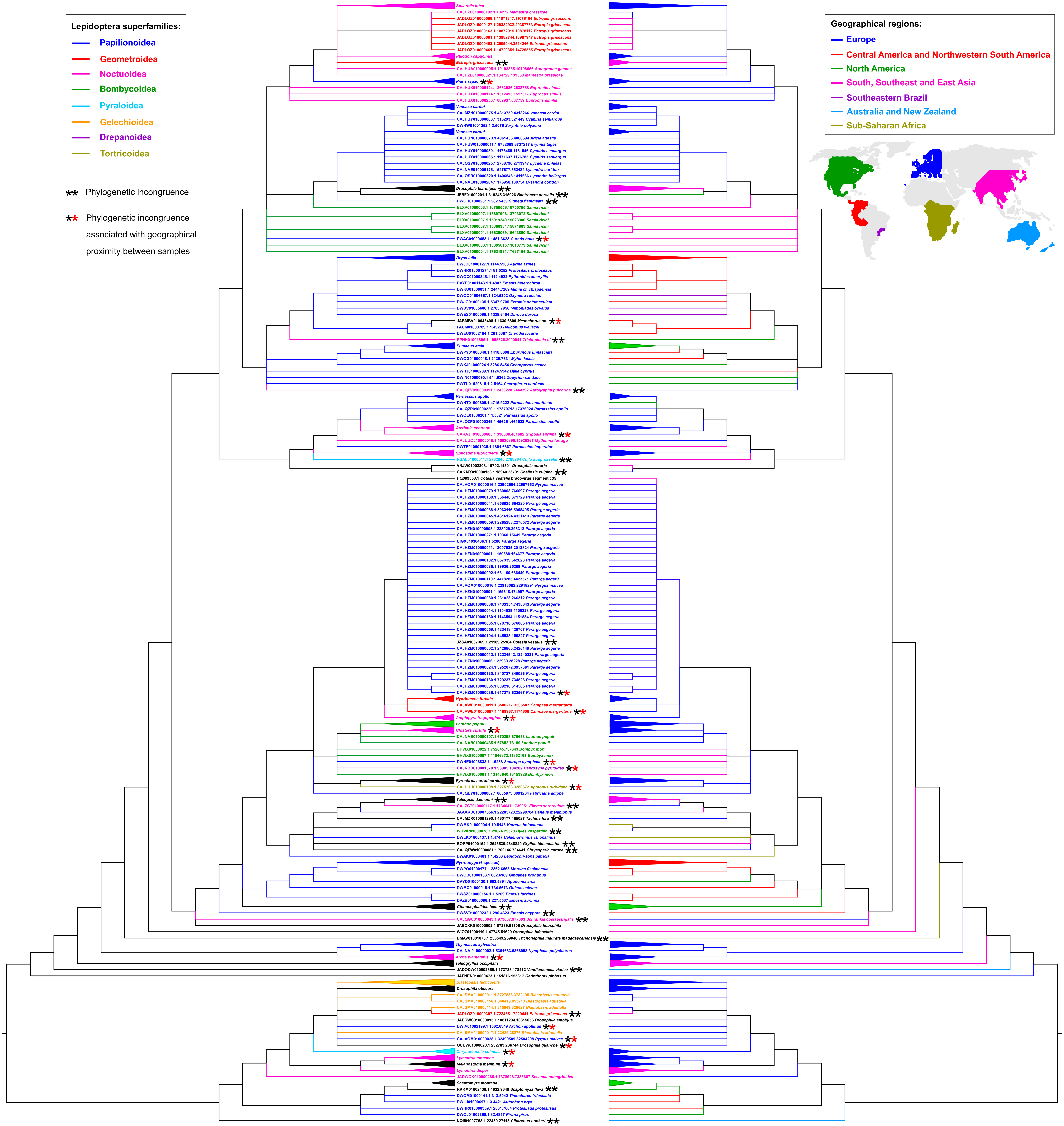

Lepidoptera superfamilies:
Papilionoidea
Geometroidea
Noctuoidea
Bombycoidea
Pyraloidea
Gelechioidea
Drepanoidea
Tortricoidea
Phylogenetic incongruence
Phylogenetic incongruence associated with geographical proximity between samples
Geographical regions:
Europe
Central America and Northwestern South America
North America
South, Southeast and East Asia
Southeastern Brazil
Australia and New Zealand
Sub-Saharan Africa

Figure S4. Graphic summary results of Blastn searches against *P. xylostella* sequencing data using Hel_c35 as a query. (A-C) Hits in the raw sequencing data from hemocytes of *P. xylostella* larvae parasitized by *C. vestalis* in three experiments. Reads mapped to Hel_c35 belonging to SRA accessions: (A) SRR11526873, (B) SRR11537818 and (C) SRR11537820. (D) Hits from searches against *P. xylostella* genome assemblies in the WGS database. (E) Reads in the raw data (SRA accession: ERR7220503) from a *P. xylostella* genome assembly (WGS accession: CAKOAA01) mapped to Hel_c35. Only the SRA run ERR7220503 from this dataset is shown as an example. See main text for further information.

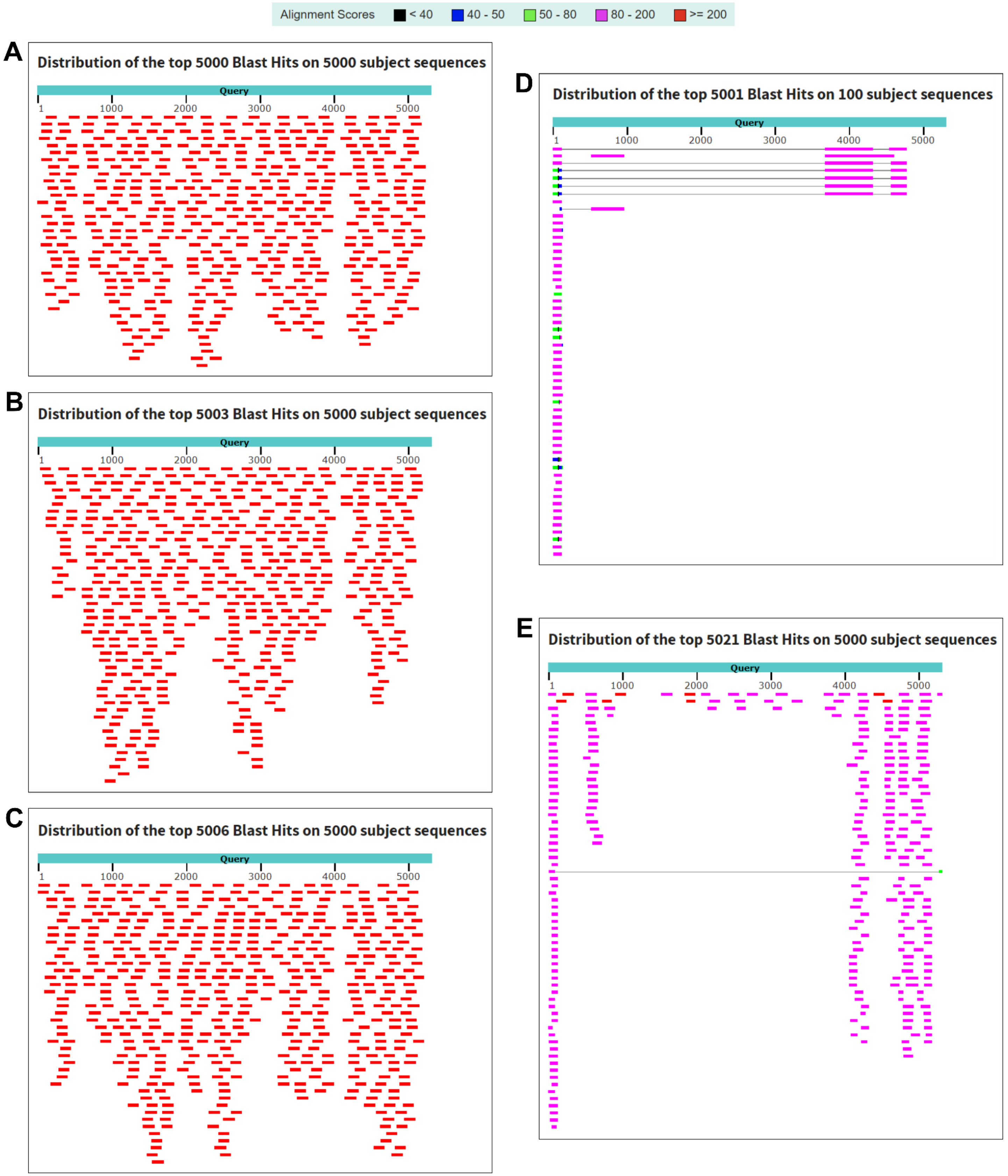

Data S1. Biopython script to only include sequences with > 70% (3705 bp) and to edit FASTA descriptions to contain only the hit accession number, the sequence match range and the species name.

```
>>> from Bio import SeqIO
>>> large_sequences = []
>>> for record in SeqIO.parse("blast_results.txt", "fasta"):
        if len(record.seq) > 3705:
                large_sequences.append(record)

>>> SeqIO.write(large_sequences, "large_seq.fasta", "fasta")
>>> clean_sequences = []
>>> for seq_record in SeqIO.parse("large_seq.fasta", "fasta"):
        seq_record.id = ((seq_record.description.split()[0])+(" ")+
                          (seq_record.description.split()[1])+(" ")+
                          (seq_record.description.split()[2]))
        seq_record.description = ("")
        clean_sequences.append(seq_record)

>>> SeqIO.write(clean_sequences, "clean_seq.fasta", "fasta")
```

Data S2. List of sequences descriptions used in the analysis, with their accession number, match range and the species name.

HQ009558.1 *Cotesia vestalis* bracovirus segment c35
CAJHZN010000006.1_22939.28228_Pararge_aegeria
CAJHZN010000001.1_159388.164677_Pararge_aegeria
CAJHZN010000001.1_169618.174907_Pararge_aegeria
CAJHZM010000138.1_366440.371729_Pararge_aegeria
CAJHZM010000104.1_145538.150827_Pararge_aegeria
CAJHZM010000080.1_261023.266312_Pararge_aegeria
CAJHZM010000059.1_423418.428707_Pararge_aegeria
CAJHZM010000045.1_4316124.4321413_Pararge_aegeria
CAJHZM010000014.1_1104039.1109328_Pararge_aegeria
CAJHZM010000011.1_2007535.2012824_Pararge_aegeria
CAJVQM010000016.1_22902664.22907953_Pyrgus_malvae
CAJHZM010000035.1_19926.25209_Pararge_aegeria
CAJHZN010000005.1_288029.293318_Pararge_aegeria
CAJHZM010000130.1_729237.734526_Pararge_aegeria
CAJHZM010000130.1_840737.846026_Pararge_aegeria
UIGX01030406.1_1.5288_Pararge_aegeria
CAJHZM010000036.1_7433354.7438643_Pararge_aegeria
CAJHZM010000035.1_609216.614505_Pararge_aegeria
CAJHZM010000035.1_617278.622567_Pararge_aegeria
CAJVQM010000016.1_22913002.22918291_Pyrgus_malvae
CAJHZM010000079.1_760808.766097_Pararge_aegeria
CAJHZM010000059.1_2265283.2270572_Pararge_aegeria
CAJHZM010000271.1_10360.15649_Pararge_aegeria
JZSA01007369.1_21189.25964_Cotesia_vestalis
CAJHZM010000038.1_5963116.5968405_Pararge_aegeria
CAJHZM010000012.1_12234942.12240231_Pararge_aegeria
CAJHZM010000002.1_2420860.2426149_Pararge_aegeria
CAJHZM010000035.1_670716.676005_Pararge_aegeria
CAJHZM010000041.1_658925.664220_Pararge_aegeria
CAJHZM010000110.1_4418285.4423571_Pararge_aegeria
CAJHZM010000130.1_1146594.1151884_Pararge_aegeria
CAJHZM010000102.1_657339.662628_Pararge_aegeria
CAJHZM010000092.1_631160.636449_Pararge_aegeria
CAJHZM010000024.1_3952072.3957361_Pararge_aegeria
CAJVWE010000011.1_3500217.3505507_Campaea_margaritaria
CAJVWE010000087.1_1169967.1174606_Campaea_margaritaria
CAJVWD010000025.1_408819.414108_Hydriomena_furcata
CAJVWD010000070.1_510413.515617_Hydriomena_furcata
CAJVWD010000025.1_377294.382499_Hydriomena_furcata
CAJVWD010000012.1_3716744.3722060_Hydriomena_furcata
CAJMZV010000185.1_996280.1001569_Amphipyra_tragopoginis
CAJMZV010000218.1_4569479.4574767_Amphipyra_tragopoginis
CAJOSM010000467.1_148944.154232_Pyrochroa_serraticornis
CAJOSM010000275.1_305597.310868_Pyrochroa_serraticornis
CAJOSM010000934.1_7793.12723_Pyrochroa_serraticornis
CAJOSL010000003.1_780653.785583_Pyrochroa_serraticornis
CAJOSM010000568.1_196017.200944_Pyrochroa_serraticornis
BHWX01000001.1_13148640.13153928_Bombyx_mori
DWHE01006833.1_1.5238_Satarupa_nymphalis
CAJNAB010000311.1_202758.208045_Laothoe_populi
CAJNAB010000435.1_67852.73189_Laothoe_populi
BHWX01000007.1_11846872.11852161_Bombyx_mori
CAJNAB010000107.1_670398.675633_Laothoe_populi
CAJNAB010000260.1_341534.346834_Laothoe_populi
CAJRBD010001370.1_98905.104202_Habrosyne_pyritoides
CAJQEY010000087.1_6085973.6091264_Fabriciana_adippe
CAJNAB010000869.1_72668.77560_Laothoe_populi
CAJQFY010000135.1_335220.340495_Clostera_curtula
CAJQFY010000347.1_1.5279_Clostera_curtula
CAJQFY010001131.1_62176.67434_Clostera_curtula
BHWX01000022.1_752045.757343_Bombyx_mori
CAJNAB010000154.1_1044455.1049578_Laothoe_populi
CAJNAB010001387.1_170726.175895_Laothoe_populi
CAJHUU010000108.1_3275763.3280672_Apotomis_turbidana
DWQB01000133.1_862.6189_Gindanes_brontinus
DVYD01000130.1_683.5891_Apodemia_ares
DWEO01003961.1_926.5870_Pyrrhopyge_telassa
DVQA01000780.1_1045.5981_Pyrrhopyge_sergius
DWOT01000734.1_76.5034_Pyrrhopyge_hadassa

DVQD01000579.1_78.4419_Pyrrhopyge_kelita
DWEP01000053.1_1589.6485_Pyrrhopyge_crida
DWSZ010000156.1_1.5209_Emesis_lacrines
CAJZCT010000117.1_1734641.1739951_Eilema_sororculum
DWLK01000137.1_1.4747_Celaenorrhinus_cf._opalinus
DWMK01000004.1_19.5148_Katreus_holocausta
DWFE01002627.1_226.5042_Pyrrhopyge_pelota
NLCU02019152.1_17889.23172_Teleopsis_dalmanni
JACTOK010041713.1_346.5639_Teleopsis_dalmanni
JACTOK010037361.1_40126.45029_Teleopsis_dalmanni
JACTOK010035260.1_62.5356_Teleopsis_dalmanni
DWPO01000177.1_2362.6863_Morvina_fissimacula
DWMC01000015.1_734.5673_Ouleus_salvina
DVZB010000096.1_227.5537_Emesis_aurimna
WUWR01000078.1_21074.25328_Hyles_vespertilio
DWSV010000232.1_290.4623_Emesis_ocypore
CAKAJF010000605.1_396300.401682_Griposia_aprilina
DWQE01036201.1_1.5321_Parnassius_apollo
DWTE010001035.1_1801.6867_Parnassius_imperator
DWHT01000605.1_4715.9222_Parnassius_smintheus
CAJOSY010001674.1_384948.390327_Atethmia_centrago
CAJOSY010000266.1_43501.48893_Atethmia_centrago
CAJOSY010000707.1_37529.42796_Atethmia_centrago
CAJOSY010001674.1_347256.352311_Atethmia_centrago
CAJQZP010000349.1_456251.461622_Parnassius_apollo
CAJQZP010001011.1_5021097.5026488_Parnassius_apollo
CAJQZP010000971.1_1549.6922_Parnassius_apollo
CAKAIX010000158.1_18940.23791_Cheilosia_vulpina
CAJUUQ010000015.1_15920890.15926267_Mythimna_ferrago
RSAL01000011.1_2782940.2788264_Chilo_suppressalis
CAJNAL010000544.1_199300.204585_Spilosoma_lubricipeda
CAJNAL010000084.1_2628222.2633568_Spilosoma_lubricipeda
CAJNAL010000037.1_461673.466985_Spilosoma_lubricipeda
CAJQZP010000220.1_17370713.17376024_Parnassius_apollo
DWKJ01000024.1_3286.8454_Cecropterus_casica
DWOG01000018.1_2139.7331_Mylon_lassia
DWPY01000040.1_1418.6608_Eburuncus_unifasciata
DWAC01000453.1_1451.6623_Curetis_bulis
DWQQ01006667.1_124.5302_Oxynetra_roscius
DWES01000095.1_1328.6454_Duroca_duroca
DVYP01001143.1_1.4807_Emesis_heterochroa
DWEU01002184.1_201.5367_Charidia_lucaria
DWHR010001274.1_81.5252_Protesilaus_protesilaus
DWJD01000127.1_1144.5908_Aurina_azines
DWKU01000031.1_2444.7269_Mimia_cf._chiapaensis
DWQC01000345.1_112.4922_Pythonides_amaryllis
DWIN01000090.1_544.5362_Zopyrion_sandace
DWDV01005608.1_2783.7956_Mimoniades_ocyalus
DWHJ01000209.1_1124.5942_Dalla_cyprius
FAUM01003789.1_1.4923_Heliconius_wallacei
JABMBV010043498.1_1630.6800_Mesochorus_sp.
CAJQFV010000391.1_2439220.2444392_Autographa_pulchrina
DWOH01000281.1_282.5439_Signeta_flammeata
DWHW01001352.1_2.5076_Zerynthia_polyxena
CAJHUW010000011.1_6732089.6737217_Erynnis_tages
CAJHUY010000088.1_316293.321449_Cyaniris_semiargus
CAJHUY010000030.1_1176489.1181646_Cyaniris_semiargus
CAJNAE010000125.1_547677.552484_Lysandra_coridon
CAJOSV010000025.1_2708796.2713947_Lycaena_phlaeas
CAJNAE010000284.1_175958.180754_Lysandra_coridon
CAJHUN010000073.1_4061456.4066594_Aricia_agestis
CAJHUY010000065.1_1171637.1176785_Cyaniris_semiargus
CAJOSR010000320.1_1406546.1411686_Lysandra_bellargus
CAJMZN010000024.1_1507394.1512552_Vanessa_cardui
CAJMZN010000124.1_3678898.3684055_Vanessa_cardui
JFBF01000201.1_310245.315026_Bactrocera_dorsalis
BLXV01000007.1_15866984.15871803_Samia_ricini
DWJG01000135.1_5347.9705_Ectomis_octomaculata
BLXV01000001.1_16639069.16643890_Samia_ricini
BLXV01000007.1_15019349.15023900_Samia_ricini
BLXV01000007.1_13697906.13703072_Samia_ricini
BLXV01000004.1_17631991.17637154_Samia_ricini
BLXV01000003.1_13005615.13010779_Samia_ricini
DWTU01020815.1_2.5164_Cecropterus_confusis

PPHH01001895.1_1995326.2000041_Trichoplusia_ni
CAJMZN010000221.1_54458.59610_Vanessa_cardui
CAJMZN010000118.1_342983.348103_Vanessa_cardui
SZUW01001905.1_136961.141302_Drosophila_biarmipes
SZUW01001905.1_119326.123659_Drosophila_biarmipes
SZUW01001905.1_128145.132475_Drosophila_biarmipes
SZUW01001905.1_123735.128069_Drosophila_biarmipes
SZUW01001905.1_132551.136885_Drosophila_biarmipes
SZUW01001905.1_114917.119250_Drosophila_biarmipes
SZUW01001905.1_141332.145691_Drosophila_biarmipes
BLXV01000003.1_10750556.10755705_Samia_ricini
JADLOZ010000452.1_2509044.2514246_Ectropis_grisescens
JADLOZ010000461.1_14720351.14725555_Ectropis_grisescens
JADLOZ010000163.1_10872915.10878112_Ectropis_grisescens
JADLOZ010000001.1_13982744.13987947_Ectropis_grisescens
CAJHUX010000250.1_682937.687759_Euproctis_similis
CAJHUX010000124.1_2633936.2638758_Euproctis_similis
CAJHUX010000174.1_1512495.1517317_Euproctis_similis
CAJHZL010000021.1_134729.139550_Mamestra_brassicae
CAJHZL010000102.1_1.4272_Mamestra_brassicae
CAJHUA010000005.1_10193835.10198656_Autographa_gamma
CAJHWT010000024.1_864030.868851_Pieris_rapae
CAJHWT010000025.1_8267413.8272232_Pieris_rapae
CAJHWT010000037.1_2774121.2778939_Pieris_rapae
CAJZHO010000673.1_459067.463887_Spilarctia_lutea
CAJZHO010000165.1_1345674.1350495_Spilarctia_lutea
CAJZHO010000105.1_881927.886748_Spilarctia_lutea
CAJZHO010000219.1_260539.265361_Spilarctia_lutea
CAJZHO010000021.1_867128.871949_Spilarctia_lutea
CAJZHO010000673.1_453918.458739_Spilarctia_lutea
CAJZHO010000143.1_1190670.1195491_Spilarctia_lutea
JADLOZ010000086.1_11071347.11076164_Ectropis_grisescens
JADLOZ010000127.1_29382932.29387733_Ectropis_grisescens
CAJZHO010000233.1_432824.437645_Spilarctia_lutea
CAJZHO010000266.1_548611.553432_Spilarctia_lutea
CAJZHO010000274.1_1798316.1803137_Spilarctia_lutea
CAJZHO010000256.1_664353.669174_Spilarctia_lutea
CAJZBM010000107.1_62863.67683_Ptilodon_capucinus
CAJZBM010000088.1_746415.751235_Ptilodon_capucinus
CAJZBM010000074.1_502408.507228_Ptilodon_capucinus
CAJZBM010000044.1_1818003.1822823_Ptilodon_capucinus
JADLOZ010000416.1_12189676.12194702_Ectropis_grisescens
JADLOZ010000492.1_6103834.6108866_Ectropis_grisescens
JAHESG010000032.1_186837.191985_Dryas_iulia
JAHESG010000017.1_6164655.6169460_Dryas_iulia
JAHESG010000029.1_7559667.7564471_Dryas_iulia
JAHESG010000004.1_15479610.15484420_Dryas_iulia
JAHESG010000016.1_3595041.3599846_Dryas_iulia
JAHESG010000029.1_3085886.3090704_Dryas_iulia
JAHESG010000009.1_9945615.9950420_Dryas_iulia
CAJMZN010000075.1_4313759.4318266_Vanessa_cardui
CAJQFW010000081.1_700146.704641_Chrysoperla_carnea
CAJMZR010001290.1_460177.465027_Tachina_fera
DWAK01008481.1_1.4253_Lepidochrysops_patricia
JAFELO010000258.1_750129.755321_Eumaeus_atala
JAFELO010000541.1_77968.82990_Eumaeus_atala
JAFELO010000139.1_388908.393951_Eumaeus_atala
JAFELO010001003.1_188367.193567_Eumaeus_atala
JAFELO010000370.1_1179360.1184501_Eumaeus_atala
JAFELO010000151.1_120296.125456_Eumaeus_atala
JAFELO010001549.1_174743.179937_Eumaeus_atala
CAJQGC010000043.1_973037.977303_Schrankia_costaestrigalis
JAECXK010000002.1_87239.91306_Drosophila_ficusphila
JAAAKD010007556.1_22285728.22290784_Danaus_melanippus
VNJW01002308.1_9702.14301_Drosophila_auraria
QVAO01001492.1_48901893.48906517_Ctenocephalides_felis
QVAO01000696.1_15497.20117_Ctenocephalides_felis
QVAO01001492.1_52438176.52442793_Ctenocephalides_felis
QVAO01002597.1_29873.34493_Ctenocephalides_felis
QVAO01001409.1_9024.13640_Ctenocephalides_felis
CAJVQL010000107.1_930262.935515_Thymelicus_sylvestris
CAJVQL010000166.1_2342372.2347626_Thymelicus_sylvestris
CAJVQL010000077.1_2140588.2145740_Thymelicus_sylvestris
CAJVQL010000077.1_2145744.2150856_Thymelicus_sylvestris

CAJVQL010000007.1_627957.633205_Thymelicus_sylvestris
CAJVQL010000185.1_2083593.2088691_Thymelicus_sylvestris
CAJNAI010000002.1_5361683.5366998_Nymphalis_polychloros
CADEBC010000506.1_2189273.2194586_Arctia_plantaginis
CADEBD010000226.1_11524308.11529654_Arctia_plantaginis
CADEBD010000226.1_5511712.5517026_Arctia_plantaginis
BMAV01001078.1_255549.259048_Trichonephila_inaurata_madagascariensis
BLKR01001327.1_272646.277539_Teleogryllus_occipitalis
BLKR01000082.1_620997.625590_Teleogryllus_occipitalis
BLKR01001662.1_236479.241317_Teleogryllus_occipitalis
BLKR01000118.1_545117.549975_Teleogryllus_occipitalis
WIOZ01000119.1_47745.51620_Drosophila_bifasciata
BOPP01000152.1_2643530.2648840_Gryllus_bimaculatus
JADODW010002850.1_173738.178412_Vandiemenella_viatica
JAFNEN010000473.1_151816.155317_Oedothorax_gibbosus
DWOJ01002358.1_82.4857_Piruna_pirus
DWHR010000359.1_2831.7604_Protesilaus_protesilaus
CAJHUO010000233.1_1599422.1604185_Blastobasis_lacticolella
CAJHUO010000267.1_2646904.2651683_Blastobasis_lacticolella
DWIA01002199.1_1562.6349_Archon_apollinus
CAJHUO010000037.1_3811342.3816132_Blastobasis_lacticolella
CAJHUO010000261.1_1929974.1934751_Blastobasis_lacticolella
CAJHUO010000046.1_898348.903124_Blastobasis_lacticolella
CAJHUO010000427.1_199617.204394_Blastobasis_lacticolella
CAJHUO010000318.1_112116.116892_Blastobasis_lacticolella
CAJHUO010000318.1_123543.128316_Blastobasis_lacticolella
CAJHUO010000345.1_691171.695944_Blastobasis_lacticolella
CAJHUO010000401.1_1220232.1225032_Blastobasis_lacticolella
CAJHUO010000228.1_159363.164129_Blastobasis_lacticolella
CAJHUO010000331.1_164805.169582_Blastobasis_lacticolella
CAJHUO010000118.1_176842.181594_Blastobasis_lacticolella
CAJHUO010000212.1_73386.78146_Blastobasis_lacticolella
CAJVQM010000028.1_32499509.32504298_Pyrgus_malvae
CAJHUO010000203.1_1777648.1782415_Blastobasis_lacticolella
CAJSMA010000017.1_23489.28276_Blastobasis_adustella
CAJSMA010000011.1_3727558.3732195_Blastobasis_adustella
CAJSMA010000156.1_848419.853213_Blastobasis_adustella
JADLOZ010000397.1_7224651.7229441_Ectropis_grisescens
CAJSMA010000114.1_315849.320627_Blastobasis_adustella
BDQP01000130.1_301209.305884_Drosophila_obscura
JAECWW010000026.1_245028.249259_Drosophila_obscura
CAJUUO010000047.1_3826225.3831009_Chrysoteuchia_culmella
CAJUUO010000001.1_1104634.1109407_Chrysoteuchia_culmella
JAECWW010000075.1_438317.443049_Drosophila_obscura
CAJHZW010001009.1_110301.115094_Lymantria_monacha
CAJHZW010000306.1_1864909.1869687_Lymantria_monacha
CAJHZW010000292.1_841817.846553_Lymantria_monacha
CAJHZW010000280.1_101693.106468_Lymantria_monacha
JAFEKU010001978.1_7197.11978_Lymantria_dispar
JAFEKT010001198.1_1395550.1400311_Lymantria_dispar
JAFEKT010002896.1_71452.76223_Lymantria_dispar
CAJHZW010001466.1_82990.87783_Lymantria_monacha
CAJHZW010000168.1_160361.165147_Lymantria_monacha
JAFEKT010001681.1_858485.863270_Lymantria_dispar
JAFEKU010001203.1_63466.68240_Lymantria_dispar
JAECWS010000095.1_10811294.10815056_Drosophila_ambigua
DWOM01000141.1_313.5042_Timochares_trifasciata
DWLJ01000697.1_3.4421_Autochton_oryx
JAEIGR010000030.1_263589.268325_Scaptomyza_montana
JAEIGR010000013.1_846018.850751_Scaptomyza_montana
RKRM01002430.1_4632.9349_Scaptomyza_flava
CAJZBV010000028.1_2548868.2553642_Melanostoma_mellinum
CAJZBV010000338.1_234267.239006_Melanostoma_mellinum
CAJZBV010000077.1_1060277.1065056_Melanostoma_mellinum
JADWQK010000266.1_7378926.7383667_Sesamia_nonagrioides
OUUW01000028.1_232789.236744_Drosophila_guanche
NQII01007758.1_22450.27113_Clitarchus_hookeri
